# Characterization of Lipid and Lipid Droplet Metabolism in Human HCC

**DOI:** 10.3390/cells8050512

**Published:** 2019-05-27

**Authors:** Nikolaus Berndt, Johannes Eckstein, Niklas Heucke, Robert Gajowski, Martin Stockmann, David Meierhofer, Hermann-Georg Holzhütter

**Affiliations:** 1 Charité—Universitätsmedizin Berlin, corporate member of Freie Universität Berlin, Humboldt-Universität zu Berlin, and Berlin Institute of Health, Institute of Biochemistry, Charitéplatz 1, 10117 Berlin, Germany; johannes.eckstein@charite.de (J.E.); hergo@charite.de (H.-G.H.); 2 Charité—Universitätsmedizin Berlin, corporate member of Freie Universität Berlin, Humboldt-Universität zu Berlin, and Berlin Institute of Health, Institute for Imaging Science and Computational Modelling in Cardiovascular Medicine, Augustenburger Platz 1, 13353 Berlin, Germany; 3 Charité—Universitätsmedizin Berlin, corporate member of Freie Universität Berlin, Humboldt-Universität zu Berlin, and Berlin Institute of Health, Department of Surgery, Augustenburger Platz 1, 13353 Berlin, Germany; niklas.heucke@charite.de (N.H.); martin.stockmann@charite.de (M.S.); 4 Max Planck Institute of Molecular Genetics, Mass Spectroscopy Facility, Ihnestraße 63–73, 14195 Berlin, Germany; gajowski@molgen.mpg.de (R.G.); Meierhof@molgen.mpg.de (D.M.)

**Keywords:** tumor metabolism, lipid droplet metabolism, mathematical model, kinetic modeling, liver, hepatocellular carcinoma

## Abstract

Human hepatocellular carcinoma (HCC) is the most common type of primary liver cancer in adults and the most common cause of death in people with cirrhosis. While previous metabolic studies of HCC have mainly focused on the glucose metabolism (Warburg effect), less attention has been paid to tumor-specific features of the lipid metabolism. Here, we applied a computational approach to analyze major pathways of fatty acid utilization in individual HCC. To this end, we used protein intensity profiles of eleven human HCCs to parameterize tumor-specific kinetic models of cellular lipid metabolism including formation, enlargement, and degradation of lipid droplets (LDs). Our analysis reveals significant inter-tumor differences in the lipid metabolism. The majority of HCCs show a reduced uptake of fatty acids and decreased rate of β-oxidation, however, some HCCs display a completely different metabolic phenotype characterized by high rates of β-oxidation. Despite reduced fatty acid uptake in the majority of HCCs, the content of triacylglycerol is significantly enlarged compared to the tumor-adjacent tissue. This is due to tumor-specific expression profiles of regulatory proteins decorating the surface of LDs and controlling their turnover. Our simulations suggest that HCCs characterized by a very high content of triglycerides comprise regulatory peculiarities that render them susceptible to selective drug targeting without affecting healthy tissue.

## 1. Introduction

Hepatocellular carcinoma (HCC) is the fifth most common cancer and the third most common cause of cancer-related deaths in the world [1]. The incidence of HCC in Europe and the United States is constantly rising, turning HCC into a pivotal threat to general health. Most cases of HCC develop on a pre-existing chronic liver disease, usually due to hepatitis C virus (HCV), hepatitis B virus (HBV), or alcohol [2]. In addition, there is an increasing prevalence of nonalcoholic fatty liver disease (NAFLD) embracing a disease continuum that starts with accumulation of triacylglycerol (TAG) in the liver (hepatic steatosis) and potentially proceeds to nonalcoholic steatohepatitis (NASH), and subsequently liver cirrhosis, a scenario which has been implicated as an additional important risk factor for the development of HCC [3].

Alterations in cellular metabolism are hallmarks of cancer. Various works suggest that lipid and glucose metabolism take an active role in hepatocellular carcinogenesis. NAFLD is characterized by an excessive accumulation of lipids in the liver due to enhanced levels of circulating free fatty acids (FFAs) in the blood plasma. Owing to the close relationship between NAFLD and aberrant systemic glucose and fatty acid metabolism in the metabolic syndrome [4], obesity and diabetes have been identified as independent risk factors for developing HCC [5,6].

The best-examined peculiarity of tumor metabolism is the famous Warburg effect characterized by increased glucose consumption and lactate production despite oxygen availability [7,8]. The molecular basis for these alterations in glucose utilization has been studied extensively, resulting in a plethora of proposed mechanisms with almost every glycolytic enzyme held accountable for the Warburg effect (for a review see [9]). In contrast, tumor-specific changes in other metabolic pathways are less clear. Especially, the contribution of the fatty acid metabolism is obscure despite the fact that hepatic lipid overload and development of HCC are closely related [5,6]. Several studies have shown that the uptake and the metabolization of FFAs correlate with tumor proliferation rates (see [7] and references within).

In general, all metabolic pathways that are active in the healthy liver also operate in HCC, however, pathway activities are adapted to the specific environment of the tumor and the specific objectives of the tumor: survival and growth. While normal liver cells have to ensure homeostatic self-maintenance, HCC cells face the challenge of accumulating enough building blocks to ensure the synthesis of the biomass required for proliferation. Currently, the fate of the FFAs taken up by HCC and the contribution of HCC to systemic liver functions, such as synthesis of ketone body and very low-density lipoprotein (VLDL), is not well understood. Although cancerous tissue is often undersupplied with nutrients and oxygen due to a general hypo-vascular environment, HCC is a hyper-vascular tumor [8]. If vascularization is not a limiting factor for substrate supply, it is plausible to assume that the uptake of FFAs by the tumor should be increased to meet the excess demand for biomass synthesis. On the other hand, it is also plausible that selfish cancer cells strive to reduce or even abolish the systemic supply with ketone bodies and VLDL particles, thereby saving fatty acids compared to healthy liver cells. Cancer cells cultured under normoxic conditions usually contain large amounts of lipid droplets (LDs), but the LD content can also be increased in response to hypoxia [9,10]. Until recently, it was unclear to what extend HCC relies on β-oxidation for ATP production. It is well established that tumors may either use glucose or fatty acids as main source of ATP production (see [11] and references within). 

The transient storage of TAG in LDs is not simply the consequence of an imbalance between the uptake of FFAs and their utilization for the generation of ATP or the synthesis of other biomolecules (membrane lipids, ketone bodies, cholesterol, VLDL, etc.), although this has been often claimed. Formation, growth, and degradation of LDs are tightly controlled by numerous regulatory surface proteins (RSPs), such as perilipins, CGI-58, ATGL (adipocyte triglyceride lipase), and the fusion protein FSP-27. The abundance and activity of these RSPs have a great impact on the efficiency with which TAG can be stored [1]. For example, the overexpression of the LD-associated lipase ATGL in the liver may increase lipolysis of LDs and thus prevent hepatic steatosis [12]. In contrast, the loss of ATGL in the liver may impair fatty acid oxidation and PPAR-α target gene expression, thus causing progressive hepatic steatosis [13,14]. Intriguingly, there exists a large cell-to-cell variability in the amount and the size distribution of LDs in hepatocytes and cancer cells [15,16] suggesting large variations in the regulation of the LD metabolism in individual HCC and in different zones of the same tumor.

The observation that changes in the expression level of a single RSP may have a large impact on the cellular LD pool inspired us to develop a kinetic model of LD metabolism in hepatocytes, which can be used to predict cellular changes in the main pathways of lipid metabolism elicited by cell-specific changes in the expression level of RSPs and metabolic enzymes [17]. The model has been validated by comparing model predictions with the outcome of a multitude of knockdown or overexpression studies of individual RSPs in the liver. The main finding of our previous model simulations was that minor cell-to-cell variations in the expression level of RSPs and metabolic enzymes in hepatocytes might give rise to substantial variations in the size distribution of LDs. The benefit of studying the in vivo metabolism of tissues by means of validated mechanistic models is twofold. First, it allows tracing, in a time-dependent manner, the concentration changes of each single metabolite and the flux changes through each single biochemical reaction in response to changes in the external conditions defined by the concentration of nutrients, oxygen, and hormones. This is an exquisite situation, which can hardly be achieved in experiments because of high ethical hurdles and severe technical and economical restrictions. Second, computational models allow "what-if analyses" to be conducted in order to anticipate the most likely metabolic response of the cell to the inhibition or activation of individual enzymes and transporters. Computational models are valuable research tools because of their capacity for generating testable hypotheses, and therefore guiding further experimentation.

In view of contradictory findings and relatively rare data on lipid metabolism in HCC, the goal of this work was to apply a kinetic model of LD metabolism in hepatocytes in order to investigate the lipid metabolism in human HCCs. We used mass spectrometry resolved protein abundances to calibrate the activities of RSPs and metabolic enzymes. On the basis of these tumor-specific instantiations of the generic model, we computed the cellular distribution of LDs in the presence of normal and elevated FFA concentrations in the plasma. Furthermore, we carried out a dynamic control analysis to assess the regulatory impact of single metabolic enzymes and RSPs on lipid accumulation in HCC. Applying this approach to eleven human HCCs revealed the existence of strikingly different metabolic phenotypes with respect to TAG metabolism. 

## 2. Materials and Methods

### 2.1. Kinetic Model of Hepatic LD Metabolism

**Figure 1:**
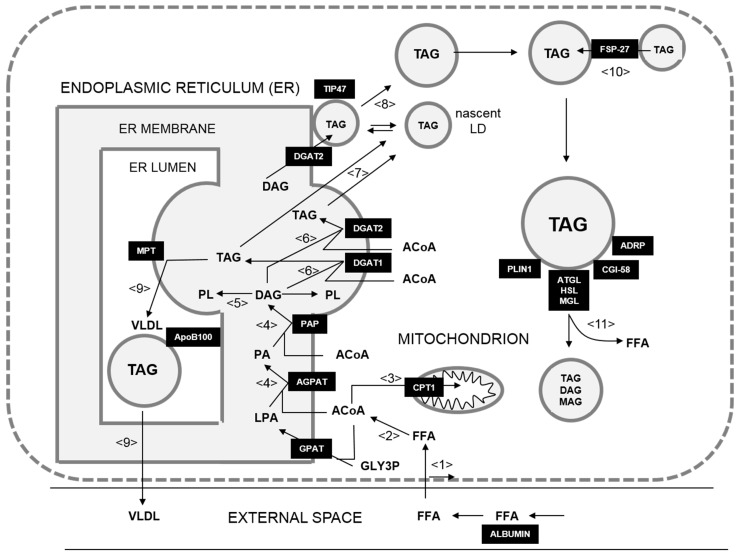
Schematic representation of the LD model. <1> Uptake of FFAs; <2> activation of FFAs; <3> uptake of FFAs into mitochondria for β-oxidation); <4> esterification of FFAs to LPA, PA, and dephosphorylation of PA to DAG; <5> phospholipid synthesis; <6> synthesis of TAG; <6> formation of TAG; <7> packing of TAG (formed by DGAT1 or DGAT2) into nascent LDs; <8> filling of LDs with TAG formed by DGAT2; <9> synthesis and secretion of VLDL; <10> TAG transfer between LDs; <11> lipolysis of LDs. Abbreviations: ACoA, acyl-coenzyme A; ADRP, adipose differentiation-related protein; AGPAT, acylglycerolphosphate acyltransferase (EC 2.3.1.51); ApoB100, apolipoprotein B100; ATGL, adipocyte triglyceride lipase (EC 3.1.1.3); CGI-58, comparative gene identification-58 protein; CPT1, carnitine palmitoyltransferase 1; DAG, diacylglycerol; DGAT1(2), diacylglycerol acyltransferase 1(2) (EC 2.3.1.20); FFA, free (nonesterified) fatty acids; FSP-27, fat-specific protein 27; GLY3P, glycerol-3-phosphate; GPAT, glycerol-3-phosphate acyltransferase (EC 2.3.1.15); HSL, hormone-sensitive lipase (EC 3.1.1.79); LDs, lipid droplets; LPA, lysophosphatidic acid; MAG, monoacylglycerol; MGL, monoglycerid lipase (EC 3.1.1.23); MTP, microsomal transfer protein; PA, phosphatidic acid; PAP, phosphatidic acid phosphatase (EC 3.1.3.4); PL, phospholipids; PLIN1, perilipin 1; TAG, triacylglycerol; TIP47, tail interacting protein 47; VLDL, very low-density lipoprotein.

As a starting point, we used the kinetic model of Wallstab et al. [17], which is given in terms of a system of 283 nonlinear differential equations governing the time-dependent change of the model variables of the reaction scheme shown in Figure 1. Kinetic equations, numerical values of the fixed (tissue-independent) model parameters (e.g., binding constants), and the tissue-specific maximal activities for the normal hepatocyte and the eleven HCCs are given in Supplementary Data 1.

The metabolic part of the model comprised the uptake of FFAs from the blood plasma (see process <1> in Figure 1) where up to seven FFAs are reversibly bound to albumin with different association constants. The model included the passive diffusional uptake of FFAs and a carrier (CD36)-mediated uptake of FFAs. FFAs are activated to acyl-CoA moieties by the enzyme fatty acyl-CoA synthetase <2>. Acyl-CoA can be taken up into mitochondria by the membrane carrier carnitine palmitoyltransferase I (CPT1) <3>, where subsequent β-oxidation yields acetyl-CoA for the citric acid cycle. As the CPT1 is the rate-limiting step in the degradation of FFAs, the activity of the CPT1 was taken as the measure for the mitochondrial β-oxidation of acyl-CoA. Alternatively, acyl-CoA can be esterified with glycerol-3-phosphate to lysophosphatidic acid (LPA) and with LPA to phosphatidic acid (PA) <4>. Hydrolytic cleavage of the phosphate group from PA yields diacylglycerol (DAG), which can be converted to TAG upon esterification with another acyl-CoA <6,7>. DAG can also be utilized for the synthesis of phospholipids <5>. There are two isoforms of the diacylglycerol acyltransferases, DGAT1 and DGAT2. It was assumed that the TAG pool of the endoplasmic reticulum (ER) membrane synthesized by DGAT1 was used for the de novo assembly of both LDs and VLDL <6>. The formation of VLDL was modeled as the transfer of TAG synthesized by DGAT1 to apolipoprotein B100 (ApoB100) by the microsomal transfer protein (MTP). Availability of ApoB100 was controlled by insulin-dependent protein degradation. The synthesis of VLDL and its release into the external space were modeled as a single step <9>. DGAT2 interacted with nascent LDs and incorporated the synthesized TAG directly into these particles <8>.

The LD module of the model was comprised of the central processes controlling the dynamics of LDs and the export of VLDL. The LDs were subdivided into 30 discrete size classes with the smallest LD having a radius of 0.1 μm and the largest LD having a radius of 3 μm. The coating of LDs with RSPs had no effect on the size class. The nascent LDs formed by de novo synthesis at the ER membrane belonged to size class LD1 and were not covered by RSPs. Each LD class was represented by the total amount of the lipid species L, that is to say L = TAG, DAG, MAG (monoacylglycerol), contained in the LDs. The number of LDs in a given LD class LD_i_ was inferred from the volume of a single LD and the total amount and volume of lipid species. An increase or decrease in the amount of any lipid species L_i_ in class LD_i_ was modeled as a lipid transfer to the next higher or lower size class LD_i+1_ or LD_i-1_, respectively. Further growth of LDs could proceed in different ways. LDs could reversibly bind to the ER membrane where they were filled with additional TAG synthesized by DGAT2 <8>. Binding to the ER membrane was facilitated in the presence of TIP47 (tail interacting protein 47). As DGAT2 associated either with the ER membrane or with LDs, the filling rate in each class depended on the LD numbers. The more LD that was present in a given size class, the higher the fraction of DGAT2 involved in the TAG filling of that class and the lower the fraction of DGAT2 associated with the ER synthesizing TAG for the LD formation. It was assumed that larger LDs were less likely to be associated with ER and being filled by DGAT2. On top of the additional lipid loading at the ER, LDs could exchange their lipid content with each other, whereby the larger particle successively absorbed the smaller one <10>. The rate of lipid transfer depended on the size differences between the LDs and on the diameter of the smaller LD, and it required the presence of FSP-27 on the surface of the fusing particles <10>. The main process responsible for the shrinking of LDs was the removal of fatty acids from the lipid esters TAG, DAG, and MAG <11>. The lipase ATGL preferentially catalyzed the conversion of TAG to DAG. The enzyme had a steady basal rate but could increase its activity after co-localization with CGI-58 <11>. The hormone-sensitive lipase (HSL) was activated by hormone-dependent phosphorylation and preferentially hydrolyses DAG. The last step that resulted in the complete lipolysis of TAG was catalyzed by the monoglyceride lipase.

The generation, growth, remodeling, and degradation of the LDs was controlled by diverse RSPs and LD-associated enzymes, which could reversibly bind to the surface of LDs. Generally, the rate of each elementary step affecting the lipid content of a LD and thus its size depended on the abundance of specific RSPs and enzymes on the LD surface. The model included reversible binding of the following RSPs to the surface of the LDs: TIP47, ADRP (adipose differentiation-related protein), perilipin (PLIN) 1, CGI-58, FSP-27, ATGL, and HSL. Reversible binding of regulatory surface proteins to LDs was modeled as a time-dependent change in the fractional surface area (XLD) covered with protein X (X = ATGL, PLIN1, ADRP, TIP47). The classical PAT proteins (PLIN1, ADRP, TIP47) were treated as competitors for unoccupied regions on the LD surface. The association constants for the binding of PATs depended on the surface curvature of the LD and the fraction of unoccupied LD surface. CGI-58 was treated as the co-regulator that was recruited to the LD surface by ADRP and PLIN1. Upon phosphorylation, CGI-58 bound to ATGL and HSL, which both bound to the unoccupied LD surface. FSP-27 was recruited to the LD surface in dependence on LD surface curvature. The association constants of the RSPs were estimated by adjusting model simulations to a large asset of independent experiments [17].

Our model contained regulatory proteins (HSL, CGI-58, PLIN1) that were regulated by hormone-dependent reversible phosphorylation. Furthermore, ApoB100 degradation was insulin-sensitive. The relationship between the phosphorylation state of hormone-sensitive proteins and the plasma level of insulin and glucagon was described by a phenomenological signal function (see Supplementary Data 2).

### 2.2. Boundary Conditions

The metabolic performance of liver cells depends on the concentration profile of hormones and exchangeable metabolites in the surrounding external space. In our model simulations, we used a diurnal plasma profiles of FFAs and hormones, which are typical for a normal physiological state. To investigate the regulatory properties of the different HCC, we calculated time-averaged values of this physiological state and a state of elevated plasma FFAs due to enhanced lipolysis in adipocytes (see Supplementary Data 2).

### 2.3. Calibration of the Basic Model to Tumor-Specific Instantiations

The HCC tissues and adjacent noncancerous tissues were taken from patients undergoing curative liver resection. Label-free LC-MS/MS shotgun proteomics was used to generate quantitative protein intensity profiles of the paired tissue samples. The maximal activity of an enzyme (v_max_) is related to the protein concentration (E) by the linear relation:

(1) $$V_{\max}=k_{\mathrm{cat}}E$$

where, k_cat_ is the catalytic rate constant (or turn-over number) of the enzyme/transporter. The shotgun proteomics yields only the protein intensity ($\tilde{E}$), which depends on the flight behavior of the peptides used as the protein identifiers. Hence, the protein intensity is related to the absolute protein concentration by an unknown factor γ,

(2) $$E=\gamma \tilde{E}$$

If the maximal activity $v_{\max}^{\mathrm{ref}}$ is already known for a reference state of the metabolic system, then the v_max_ value for a different metabolic state (here the metabolic state of the tumor) can be calculated by exploiting the Equations (1) and (2):

(3) $$v_{\max}^{\mathrm{tumor}}{=v}_{\max}^{\mathrm{ref}}\frac{{\tilde{E}}^{\mathrm{tumor}}}{{\tilde{E}}^{\mathrm{ref}}}$$

Usage of relation (3) circumvents the problem of converting the protein intensities to absolute protein abundances. The vector constituted by the enzyme intensity ratios $\frac{{\tilde{E}}^{\mathrm{tumor}}}{{\tilde{E}}^{\mathrm{ref}}}$ defines the enzyme expression signature of the tissue under investigation. For the proteins, where an intensity ratio could not be defined due to missing data in the label-free quantification (LFQ) intensities, we set the intensity ratio to one. 

### 2.4. Acquisition of Tissue Samples 

HCC tissues and adjacent noncancerous tissues were collected from eleven patients, which underwent curative resection (R0) at the Department of Surgery of the Charité. Ethical approval for tissue sampling and analysis was obtained on June 26, 2015 from the ethics committee at Charité—Universitätsmedizin Berlin (EA1/140/15). The patients gave informed consent to the scientific use of their resected tissue specimen for molecular analyses. Macroscopically, the analyzed HCC tissues were graded according to the Tumor-Nodes-Metastasis classification [18]. No distant metastases or spreading to lymph nodes were found. The resected tissues were used for tumor grading according to Hamilton and Aaltonen (Eds.), 2000 [19].

### 2.5. Quantitative Proteomics of Tissue Samples

Frozen liver tissue was cut into pieces of ~10 mg with cooled tweezers and scalpel in a precooled mortar. They were transferred into screw lid vials, which contained a precooled disruption bead and 1 mL lysis buffer. Additionally, samples were kept on dry ice to prevent thawing. To avoid proteolytic activity, tissues were homogenized immediately with a FastPrep. Tissues that were homogenized under denaturing conditions with a FastPrep (three times for 60 s, 6.5 m × s^−1^) in a lysis buffer containing 1% sodium deoxycholate, 10 mM tris(2-carboxyethyl)phosphine, 40 mM chloroacetamide, and 100 mM Tris pH 8.5. The homogenates were then lysed at 95 °C for 10 min followed by 5 min sonication. The 50-µL aliquots of lysates were digested and purified using the preOmics in-stage tip kit (iST kit 96×, Martinsried, Germany). Samples were eluted sequentially in three fractions using the SDB-RPS-1 and -2 buffers [20] and the elution buffer provided by preOmics for subsequent analysis on a nanoLC-MS.

LC−MS/MS was carried out by nanoflow reverse phase liquid chromatography (Dionex Ultimate 3000, Thermo Scientific, Waltham, MA, USA) coupled online to a Q-Exactive HF Orbitrap mass spectrometer (Thermo Scientific, Waltham, MA, USA). The LC separation was performed using a PicoFrit analytical column (75 μm ID × 55 cm long, 15 µm Tip ID (New Objectives, Woburn, MA, USA) in-house packed with 3-µm C18 resin (Reprosil-AQ Pur, Dr. Maisch, Ammerbuch-Entringen, Germany), as reported previously [20]. Briefly, peptides were eluted using a gradient from 3.8 to 50% solvent B in solvent A over 121 min at 266 nL per minute flow rate. Solvent A was 0.1% formic acid and solvent B was 79.9% acetonitrile, 20% water, 0.1% formic acid. Nanoelectrospray was generated by applying 3.5 kV. A cycle of one full Fourier transformation scan mass spectrum (300−1750 m/z, resolution of 60,000 at m/z 200, AGC target 1e6) was followed by 12 data-dependent MS/MS scans (resolution of 30,000, AGC target 5e5) with a normalized collision energy of 25 eV. In order to avoid repeated sequencing of the same peptides a dynamic exclusion window of 30 s was used. In addition, only the peptide charge states from two to eight were sequenced.

Raw MS data were processed using MaxQuant software (1.5.7.4; Max-Planck Institute of Biochemistry, Computational Systems Biochemistry, Martinsried, Germany) [21] with the Andromeda search engine [22] and the human UniProtKB with 70,228 entries released in February 2016. A false discovery rate of 0.01 for proteins and peptides, a minimum peptide length of seven amino acids, a mass tolerance of 4.5 ppm for precursor and 20 ppm for fragment ions were required. A maximum of two missed cleavages was allowed for the tryptic digest. Cysteine carbamidomethylation was set as fixed modification, while N-terminal acetylation and methionine oxidation were set as variable modifications.

### 2.6. Mapping of Protein Intensities Onto Model Processes

In total, the proteomics yielded signals for 6502 protein groups. From this total set, we selected a subset of 43 proteins, which were assigned to the 17 model reactions mediated by enzymes and transporters. Several protein intensities, (P1,…,Pn), were assigned to a model reaction using the MaxQuant software and we calculated a mean protein intensity.

(4) $$<P>=\sum_{i=1}^{n}a_{i}P_{i}$$

with the constraints

(5) $$\sum_{i=1}^{n}a_{i}=1,\sum_{i=1}^{n}({<P>-P}_{i}{)}^{2}\to \mathrm{miniumum}!$$

According to the condition (4), the coefficients used for the linear combination of the individual are chosen such that they attain a minimum.

The percentage of enzymes, transporters, and RSPs for which at least one protein intensity value was available from the proteomics analysis was 70%, with the lowest coverage of 48% and the highest coverage of 81%. In order to fill the gaps, we applied a statistical imputation method, which estimates missing values of a given target protein based on the expression profiles of several other predictor proteins with sufficiently similar intensity profiles. This method makes the assumption that either genes or proteins are regulated dependently and that highly correlated expression behaviors are normally observed with co-regulated genes.

First, we compiled a list of potential predictor proteins for which measured intensities were available for all eleven controls and eleven tumors. This resulted in a set of 1675 predictor proteins. Second, for a given target protein with missing protein intensity values in some tumors, we determined a group of predictor proteins exhibiting a Spearman correlation larger than a critical threshold value R_crit_ = 0.8. Linear regression analysis with each of these predictor proteins yielded estimates of the missing values. Finally, the missing intensity values were filled by the mean of the estimates obtained for all predictor proteins. The quality of the imputation was assessed by the coefficient of variation (standard deviation devised by the mean). We included in the imputation procedure only target proteins comprising at least four out of eleven experimentally determined protein intensities because otherwise the chance of randomly high correlations was too large. An example illustrating the used imputation method is shown in Supplementary Data 3. The list of measured and imputed protein intensities used for model calibration is given in Supplementary Data 4.

## 3. Results

### 3.1. Histological Characterization of Tissue Samples

All tumors were of low or moderate extension (T1–T2). Except for one case, there was no tumor invasion into the lymph nodes or vein. The degree of fibrosis in the tumor-surrounding tissue varied largely from absence of fibrosis (F = 0) to cirrhosis (F = 4).

### 3.2. Protein Intensity Profiles of Tumors and Noncancerous Tissue

First, we compared protein intensity profiles (defined through LFQ intensities, see Methods) of eleven HCCs (T1–T11) and related noncancerous adjacent tissue (C1–C11). In total, the proteomics yielded signals for 6502 protein groups. We also compared protein intensity profiles of the 43 proteins, which we used to calibrate the maximal activities and enzymes and transporters occurring in the metabolic model. The similarity analysis was based on pairwise Pearson’s and Spearman’s correlation coefficients (which is more informative than Pearson’s correlation coefficient if the statistical distribution of the data deviates from the normal one). In this correlation analysis, we skipped imputed values, i.e., only the experimentally determined intensities were included.

**Figure 2:**
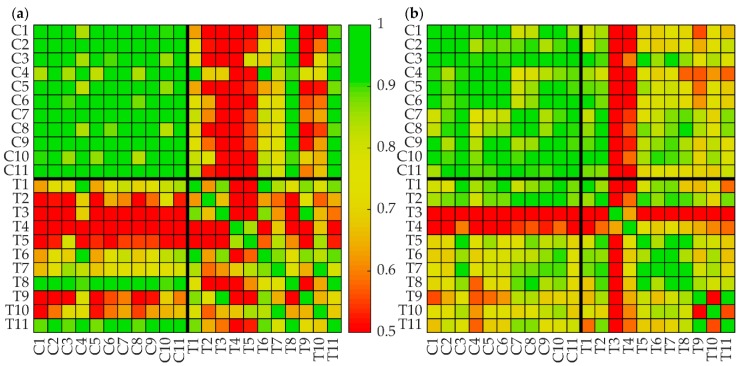
Heatmaps of correlation coefficients of protein intensity profiles. (**a**) Pearson’s correlation of the intensity profiles of 43 proteins included in the model, (**b**) Spearman’s correlation of the intensity profiles of 43 proteins included in the model. HCC: T1–T11, adjacent noncancerous tissue: C1–C11.

Figure 2 reveals consistently higher similarities among the protein intensity profiles of the noncancerous tissue samples irrespective of varying degrees of fibrosis of the tumor-adjacent tissue (see Table 1). For the subset of proteins included in the kinetic model the homogeneity between the control tissues was R = 0.94, while the inhomogeneity between tumor tissues was reflected by a much smaller R of 0.65. Thus, metabolic deviations of the noncancerous tissue from normal liver tissue should be rather uniform and small as compared with metabolic differences among the tumors. 

### 3.3. Tumor-Specific Calibration of the Kinetic Model

To investigate the functional implications of tumor-specific protein abundances in the eleven human HCCs, we performed simulations under varying external conditions. For the calibration of tumor-specific model instantiations, we scaled the maximal activities of enzymes, transporters, and RSPs according to formula (3) based on the relative mean protein abundances in the tumor as compared with the control given in Supplementary Data 4. In order to obtain a full protein coverage of the model network, we substituted missing experimental data using the imputation method explained in Materials and Methods. For the two model processes associated with the proteins ATGL and CGI-58, no protein intensities could be measured by the shotgun proteomics. The relative change of protein abundances was arbitrarily put to unity, in other words, the activities of these proteins in the control and the tumor were assumed to be identical. 

Furthermore, we made the assumption that the protein intensities of the noncancerous tissue adjacent to the tumor were close to those of a normal liver tissue. In order to check how this assumption influenced the results of our computational analyses, we carried out all simulations by using in the scaling formula (3) an ensemble of reference activities, which were randomly sampled from a 10% interval around the maximal activity $v_{\max}^{\mathrm{normal}}$ of the normal liver.

### 3.4. Lipid Metabolism of Individual HCCs at Normal Plasma Profile of FFAs

**Figure 3:**
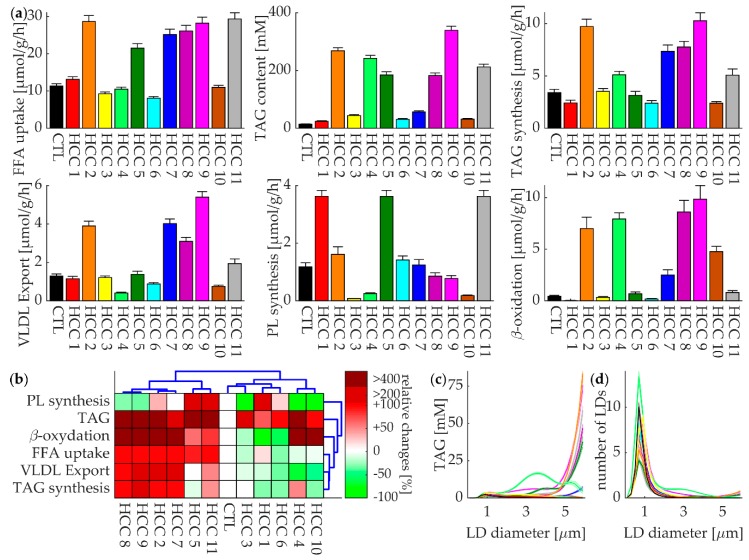
Metabolic profiles of lipid metabolism of individual HCCs based on simulated time-dependent changes of metabolites and fluxes. (**a**) 24-h mean values of six metabolic functions for the control (black column) and eleven HCCs. The error bars were obtained by randomly sampling the maximal activities of all model proteins from a 10% interval around the reference values, (**b**) clustering of the control and eleven HCCs based on similarities between their metabolic functions scaled as the percentage relative to the control. Values >100% were replaced by a cut-off value of 100% to avoid having a single function with a very large increase relative to the control dominate the cluster analysis, (**c**) 24 h average TAG content stored in LDs of different size, (**d**) 24 h average size distribution of the LDs.

The metabolic performance of the liver depends largely on the concentration profile of hormones and exchangeable metabolites in the extracellular space. We approximated the composition of the external milieu by a generic 24-h plasma profile (see Supplementary Data 2) and simulated time-dependent changes of all metabolites and fluxes covered by the model. The 24-h time-average of the simulated time courses of six important metabolic functions was used to define the metabolic profile of a tumor (Figure 3a). This analysis revealed remarkable metabolic differences among the eleven HCCs. As compared with the control tissue, the uptake rate of FFAs was reduced in four HCCs but increased more than two-fold in six HCCs. The tumors with high FFA uptake rates also had high rates of TAG synthesis, VLDL export, and β-oxidation. Two of the six tumors had increased rates of phospholipid synthesis. In contrast, the tumors without increased FFA uptake rates had decreased rates of VLDL export and TAG synthesis. Phospholipid synthesis rates were decreased in three of these tumors and three tumors had decreased rates of β-oxidation. Accumulation of TAG was increased in all tumors, with six tumors exhibiting very high TAG content. Five of these six tumors belonged to the group with increased FFA uptake rate.

Counterintuitively, some tumors (#2, #4, #8, and #9) with increased activity of β-oxidation, nevertheless, had a high TAG content. This can be explained by changes in the abundance of RSPs that can even reverse the effect elicited by an increase of the activity of the enzyme/transporter CPT1. The flux through CPT1 is not only determined by the activity of the transporter but also by the rate of β-oxidation, which in turn depends on the total energy demand of the cell. Moreover, the rapid esterification of FFAs and efficient storage in terms of TAG in LDs lowers the cytosolic level of FFAs and thus additionally limits the flux through the CPT1. Hence, taking the abundance of a single enzyme protein (here CPT1) as the only determinant of the flux strength can be completely misleading. 

We performed a cluster analysis to group the HCCs according to similarities between their 24-h average metabolic profiles. To this end, we scaled each metabolic function as percentage change with respect to the control (i.e., 50% TAG content of HCC1 in Figure 3b means a 50% increased TAG content with respect to the control). The clustering was done using the statistics and machine learning toolbox of MATLAB 2018a (The MathWorks, Inc., Natick, MA, USA) and its implementation of Ward’s minimum variance method on the Euclidean distance between the data points. This analysis supported the above argumentation with respect to the various HCCs that could be subdivided into tumors with low or high lipid turnover and differed in the activity of the FFA metabolizing pathways (Figure 3b). The "low lipid turnover" cluster was comprised of tumors that metabolically resembled the healthy hepatocyte characterized by a globally downregulated lipid metabolism. Notably, irrespective of large differences in the total number of LDs (correlating with the total TAG content), the size distribution of LDs of all tumors was similar (see Figure 3d). In tumors with very high TAG accumulation, although the LD distribution peaked at diameters around 1 µm, the majority of TAG was stored in a few very large LDs.

### 3.5. Lipid Metabolism of Individual HCCs at High Plasma Profile of FFAs

**Figure 4:**
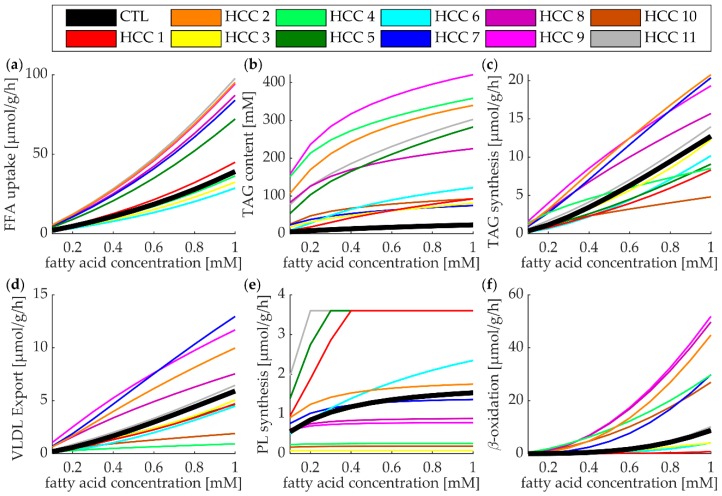
Metabolic response of HCCs and control to increasing external concentrations of FFAs. Color assignment to tumors and control is the same as in Figure 3. (**a**) Free fatty acid (FFA) uptake, (**b**) intracellular TAG content, (**c**) TAG synthesis rate, (**d**) VLDL export rate, (**e**) phospholipid (PL) synthesis rate, (**f**) rate of β-oxidation (i.e., activity of CPT1).

Next, we investigated the metabolic response of individual HCCs in response to rising plasma concentrations of FFAs. To this end, we calculated the metabolic steady state that was reached if the tumor was constantly exposed to an external FFA concentration between 0.1 mM and 1 mM (Figure 4). Generally, increasing FFA concentrations resulted in a higher activity of the metabolic functions. The ranking of tumors with respect to the absolute magnitude of a given metabolic function was preserved with increasing FFA concentration, however, in most cases the inter-tumor differences were amplified in a nonlinear fashion. This holds in particular for the β-oxidation. Whereas, at a normal diurnal plasma level of FFAs the maximal inter-tumor differences between the β-oxidation rates was about 5 μmol/g/h (see Figure 2a) and the maximal difference could exceed 40 μmol/g/h at an external FFA concentration of 1 mM.

### 3.6. The Regulatory Impact of Enzymes and RSPs on the TAG Content of Tumors

For an efficient and selective pharmacological interference with the lipid metabolism of HCC, it is important to know how sensitive the TAG content responds to the inhibition or activation of an enzyme or RSP selected as a potential drug target. Therefore, we performed control analysis by quantifying the regulatory impact of a protein on the cellular TAG content elicited by a small (10%) reduction in the activity of a given target protein:

(6) $$\Delta \mathrm{TAG}\left(\%\right)=\frac{{\mathrm{TAG}}_{\mathrm{inh}}{-\mathrm{TAG}}_{\mathrm{norm}}}{{\mathrm{TAG}}_{\mathrm{norm}}}\times 100$$

TAG_norm_ denotes the TAG content at normal protein activity and TAG_inh_ denotes the TAG content at 10% reduced protein activity. As the regulatory impact of a protein may vary considerably among the different metabolic states [24], we calculated ΔTAG at two distinct physiological states characterized by a normal plasma profile of FFAs, insulin, and glucagon ("normal plasma FFA") and a plasma profile characterized by persistently elevated concentrations of FFAs and glucagon and reduced concentrations of insulin ("high plasma FFA"). The absolute values of TAG_inh_ and TAG_norm_ are given in Supplementary Data 5.

**Figure 5:**
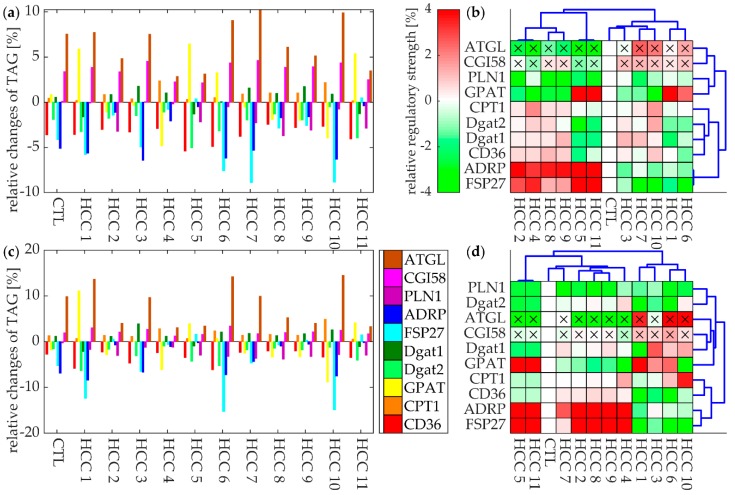
Regulatory impact of enzymes and RSPs on the TAG content. Panels in the upper or lower row show the results at normal or elevated plasma concentrations of FFAs, respectively. Clustering of tumors (x-axis) was performed according to similarities of their regulatory profiles, which are different under normal and elevated FFA plasma levels. Clustering of the regulatory proteins (y-axis) was performed according to similarities of the inhibition-induced TAG changes in different tumors. Boxes marked by an (x) depict the two proteins, where the enzymatic signature was set to unity due to completely missing protein intensity values. (**a**,**c**) Regulatory profiles of the ten most important regulatory proteins with ΔTAG > 6% in at least one tumor, (**b**,**d**) grouping of tumors according to the similarities of their regulatory profiles. Coloring indicates the size of the tumor-selective effect given by the difference ΔTAGtumor−ΔTAGcontrol.

We define the regulatory profile of the tissue by the vector of ΔTAG values for all processes included in the model. Figure 5a,c shows the regulatory profiles of the ten regulatory most relevant proteins possessing absolute ΔTAG values larger than 6% in a least one tissue. There are substantial differences among the tumors with respect to the absolute magnitude of ΔTAG values. For example, the regulatory impact of FSP-27 on the TAG content of HCC #6, #7, and #10 is about one order of magnitude larger than in HCC #4, #5, and #11. Generally, the impact of protein inhibition of the cellular TAG content was larger at an elevated plasma level of FFAs. In both physiological states, the regulatory surface protein ATGL had the largest impact on the TAG pool followed by the LD fusion-promoting protein FSP-27 and the glycerol-3-phosphate acyltransferase (GPAT) (which is responsible for the initial step of TAG synthesis). Surprisingly, the relative impact of the mitochondrial fatty acid transporter CPT1 was low in most tumors, suggesting that pharmacological activation of CPT1 should have little effect on the TAG pool of HCC in general, but could become important under high fat conditions.

In Figure 5b,c, we arranged the tumors according to similarities between their regulatory profiles and the regulatory profile of the control. This “regulatory grouping” was similar to the “functional grouping” shown in Figure 3b, suggesting that metabolically similar tumors come along with similar regulatory properties. The tumors belonging to the group with an active fatty acid metabolism are mainly controlled through ADRP and FSP-27, while the group of tumors with reduced fatty acid metabolism is mainly regulated by ATGL and CGI-58. However, there are also exemptions: HCC#4 characterized by a modest fatty acid utilization is under strong control of ATGL and HCC#7 characterized by an intense fatty acid utilization is under strong control of ADRP and FSP-27.

The coloring in Figure 5b,d highlights the tumor-selective effect, ΔTAG_tumor_−ΔTAG_control_, achieved by inhibition of the ten most important regulatory proteins. Importantly, the tumor-selective effect may be positive and negative, in other words, inhibition of one and the same protein may result in a larger increase or decrease of the TAG content in the tumor as compared with the control. For example, at a normal plasma level of FFAs, inhibition of FSP-27 is predicted to exert a large selective effect in most tumors, but in one group of tumors (#2,4,5,11) the effect is smaller than in the control, whereas, in another group of tumors (#6,7,10) the effect is stronger than in the control. A high tumor-selective regulatory effect is an important criterion for choosing those proteins as potential drug targets, which upon inhibition exert a larger effect in the tumor than in the surrounding noncancerous tissue. Thus, if the aim was to selectively lower the TAG reserve of HCC#10, our analysis suggests that inhibition of GPAT or FSP-27 should yield the largest effect.

## 4. Discussion

### 4.1. Tissue Metabolism In Vivo: Detailed Insights by Means of a Computational Approach

Assessing metabolic activities of tissues/organs in vivo is still a challenge. The method of choice consists in tracing the labeling of various metabolites after administration of nutrients such as glucose, pyruvate or glutamine, which are labeled with stable isotopes (e.g., ^13^C, ^2^H, and ^15^N). However, this method is restricted to the analysis of very few pathways that can be reached by the administered label and is not suited for monitoring metabolic fluxes over a longer period and at varying plasma profiles of nutrients. To overcome these limitations in this work, we applied an approach that is based on a detailed kinetic model of the hepatic LD metabolism. We used mass spectrometry-based protein abundance ratios between the HCC and the surrounding tissue to scale the activity of the proteins included into the model. In this way, we generated individualized models of hepatic lipid metabolism for eleven different human HCCs. Our goal was to investigate how different FFA metabolizing pathways of HCC respond to circulating FFAs and to assess the relative importance of individual proteins for the control of the tumor’s TAG content.

### 4.2. Main Finding

Our analysis suggests that different metabolic phenotype exist in human HCC, characterized by a high and low fatty acid metabolism.

### 4.3. Comparing our Findings with Known Metabolic Features of HCC

Uptake and β-oxidation of FFAs: According to our simulations, the examined individual HCCs can be grossly subdivided into two groups differing substantially in the rate of FFA uptake and the activity of FFA metabolizing pathways. This result is in agreement with the finding that subclasses of HCC are addicted to fatty acids [25]. These tumors carry an activating mutation in the *CTNNB1* gene, encoding β-catenin. Upregulation of enzymes of the β-oxidation pathway is under the control of the transcription factor PPARα targeted by ß-catenin. The importance of β-oxidation for cancer cells is not uniform. Especially for cancer cells exposed to a hypoxic environment, β-oxidation of fatty acids should only play a minor role for energy production owing to the lower ATP/O_2_ yield of fatty acids as compared with glucose [11]. In line with this, a HIF-1 mediated suppression of β-oxidation and accompanying accumulation of LDs in HCC was observed under hypoxic conditions [26]. On the other hand, it was shown for murine primary tumors that depletion of CPT1 via siRNA suppressed xenograft tumor growth and metformin responsiveness in vivo. The growth advantage of high or low rates of FFA utilization will largely depend on the specific tumor environment. Depending on the vascularization of a solid tumor, intra-tumor regions with chronic hypoxia may coexist with regions that experience only transient hypoxic episodes (cycling hypoxia) [27]. For the latter regions, the enhanced oxidation of pre-accumulated fatty acids during a re-oxygenation period could greatly facilitate tumor growth [28].

Phospholipid synthesis: Our results indicate that alterations in the capacity for phospholipid synthesis in the examined HCCs range from almost complete abolishment to marked increase. Unfortunately, there are very few experimental studies on the phospholipid metabolism in HCC. An old study found the phospholipid content of Novikoff hepatoma to be markedly lower than that of the normal rat liver [29]. This change was specific to the neoplastic fever because there was no such alteration present in the rapidly growing regenerating liver. ^32^P incorporation studies suggested that the phospholipid depletion in the hepatotomy (down to 30% of the normal) was due to a reduced de novo synthesis. A lower share of phospholipids in the total lipid pool was also found in human xenograft in nude mice [30].

VLDL export: The heterogeneous pattern of VLDL synthesis rates among individual HCCs mirrored that of the FFA uptake rates. The VLDL secretion rates primarily reflect the expression level of ApoB100 and MTP facilitating in the ER the transfer of TAG and cholesterol esters onto the nascent lipoprotein. Human gene expression data collected from four independent HCC cohorts (*n* = 941) also showed a large scatter of APOB activities, whereby, poor prognosis was consistently observed with APOB inactivation [31].

Triglyceride accumulation: An important finding of our study was that the accumulation of TAGs in HCC does not reflect the uptake rate of FFAs. The amount of TAG stored in LDs is not only determined by the balance between uptake and utilization of FFAs, but also depends on the balance between synthesis and degradation of LDs. At the extreme, complete downregulation of all LD degrading lipases would result in a steadily growing TAG pool even at a very low FFA uptake. The molecular basis for the excessive accumulation of TAG in HCC#4,5,9 and HCC#11 lies in the very high abundance of ADRP (perilipin 2). Activation of ADRP can be induced by HIF-1α during hypoxia [9] but also by HIF-2α under normoxic conditions [10]. This RSP is resident on the membrane of LDs and promotes the de novo synthesis of LDs.

Of note, the expression level of PLIN1, acting as a protective coating from the LD degrading lipases, was also markedly increased in most HCCs (#1,2,4–6,8–11), leading to few LDs with a very high TAG content. This prediction is in line with observations according to which PLIN1 resides only on very large LDs and is generally not present in liver tissue (see [32] and references therein). The inter-tumor variations in the abundance of TIP47, a perilipin involved in the control of LD grows and maturation, are much smaller as compared with variations in the abundance of ADRP. Thus, as compared with TIP47, ADRP appears to be the better marker for the TAG storing capacity of human HCC.

Roles of LD in HCC: LD content is increased in all HCCs (see Figure 3; Figure 4), raising the question of the biological benefits of high intrinsic fat accumulation. LDs in cancer cells have been implicated in a number of different functions. One such function is the protection that LDs may provide against ER stress, caused by the accumulation of misfolded proteins. LDs may alleviate ER stress by serving as a temporary depot for misfolded proteins [33] and positively affecting the elimination of misfolded proteins through proteasome regulation [11]. LDs may scavenge reactive oxygen species and sequester lipophilic anti-tumor drugs, and therefore provide HCC a remarkable drug resistance (see [34] and references within).

Targeting regulatory proteins of LD metabolism in HCC: Our control analysis for the cellular TAG content shows that different patterns of key regulatory proteins exist (Figure 5). This finding has important implications for the design of an efficient drug therapy aiming at the reduction of the TAG content in a tumor. The tumor cluster formed by a highly active fatty acid metabolism is characterized by severely increased LD content mainly regulated by ADRP and FSP-27. ADRP is responsible for shielding LDs against basal lipolysis. Targeting ADRP would thus lower the cellular TAG content by increasing the basal lipolytic rate. FSP-27 is indispensable for the fusion of LDs. Since larger LDs have a higher surface to volume ratio and thus require less phospholipids and RSPs to store a given amount of TAG, targeting FSP-27 would reduce the effectiveness of TAG storage in the tumor. However, the same strategy would lead to a more pronounced reduction in the control as compared with the cluster formed by low fatty acid metabolism. Therefore, the sensitivity of the TAG content against changes in the activity of proteins can be very different for different tumors which makes a general treatment strategy unlikely.

Limitations of the approach: The most difficult issue with the tumor-specific parameterization of the kinetic model is the definition of the "normal" control. The maximal enzyme activities of the basic model [17] were chosen to achieve the best possible concordance of model simulations with experimental data for healthy livers. However, protein activities in liver samples of an individual subject, regardless whether taken from an apparently healthy region, from the environment of a tumor or from the tumor itself, will deviate from those of the generic model defining the normal metabolic reference state. Aware of this issue and lacking anything better, we equated the protein intensity profiles of the tumor-adjacent tissue with "normal" enzyme activities. A certain justification of this setting comes from the persistently high correlation of protein intensity profiles among non-tumorous liver samples (see Figure 2). In order to assess the robustness of our findings against a possibly wrong assignment of maximal protein activities to the measured protein activities of the control, we performed a resampling procedure by replacing $v_{\max}^{\mathrm{normal}}$ of the control tissues by a value randomly chosen from a ±10% interval around $v_{\max}^{\mathrm{normal}}$. This procedure allowed putting standard deviations to the calculated values of the metabolic functions. Still, deviations larger than 10% between the maximal protein activities of control tissues and healthy tissue cannot be ruled out.

Another issue with mapping protein abundances onto molecular networks is the incomplete network coverage. In our approach, the average network coverage achieved with experimentally determined protein intensities was 70%. Label-free quantitative proteomics is meanwhile a standard method to efficiently generate genome-wide protein intensity profiles that can be used to quantify changes of protein abundances in different physiological settings. Unfortunately, a substantial fraction of data is usually missing from proteomic datasets owing to a complex combination of random and nonrandom processes [35]. Since the parametrization of kinetic network models, such as the one used in this study, requires relative protein abundances for all biochemical processes, imputation methods have to be used to fill the gaps. Among several proposed imputation approaches, those based on the local similarity of protein profiles have been shown to yield the best overall performances with respect to metrics of accuracy and robustness [36]. Therefore, we used a gap-filling approach that estimates the unknown intensity of a target protein in a tissue sample by a linear regression model representing the relationship between the measured intensities of the target protein and those in a set of sufficiently similar reference proteins. The biological plausibility of the imputation method follows from the fact that numerous genes and proteins are under control of the same gene-regulatory circuits [37]. Moreover, the reliability of the imputation method is supported by the fact that on average the coefficient of variation for the imputed values was CV = 0.63 for the controls and CV = 0.83 for the tumors

A third problem is the definition of appropriate boundary conditions. Owing to the structural alterations of the tumor-surrounding liver parenchyma (vascularization, enlargement of the Disse space in fibrotic regions, etc.), the plasma profile of metabolites and hormones that are used may be different from the profile actually sensed by the tumor. The extension of our recently published metabolic tissue model [38] to fibrotic and cirrhotic liver regions may help to include structural effects impairing the exchange of compounds between a tumor and plasma.

## 5. Conclusions

Our analysis shows that LD metabolism in HCC is heterogeneous among individual tumors, however, functional and regulatory features are highly interdependent. Especially those HCCs that are characterized by a very active fatty acid metabolism comprise regulatory peculiarities that render them susceptible to selective targeting without affecting healthy tissue.

## Figures and Tables

**Table 1:** Characteristics of tumors and adjacent noncancerous tissues. HCCs were classified according to the Tumor-Nodes-Metastasis classification system of malignant tumors [18]. T(0–4)—tumor size; N(0–3)—spread to regional lymph nodes; G(1–4)—differentiation grade; L(0–1)—invasion into lymphatic vessels; V(0–2)—invasion into vein; R(0–2)—completeness of the operation (resection boundaries free of cancer cells or not). The degree of fibrosis F(0–3) and presence of cirrhosis (F4) in the adjacent noncancerous tissue was assessed according to the Desmet and Scheuer scoring system [23].

HCC#	Gender	Age	T	N	G	L	V	R	Fibrosis	Liver Disease
1	f	70	2	-	2	0	1	0	4	Alcohol-induced cirrhosis
2	m	68	2	0 (0/1)	2	0	1	0	2	Cryptogenic fibrosis *)
3	m	67	1b	-	2	0	0	1	1	Cryptogenic fibrosis *)
4	m	80	1	-	3	0	0	0	1	Alcohol-induced hepatic fibrosis
5	f	22	1	-	1	0	0	0	0	No chronic liver disease history
6	f	67	1a	-	2	0	0	0	4	Cryptogenic cirrhosis *)
7	m	73	2	-	2	0	0	0	4	Alcohol-induced cirrhosis
8	m	71	2	-	2	0	0	0	3	Alcohol-induced advanced hepatic fibrosis
9	f	66	1b	-	2	0	0	0	4	NASH cirrhosis
10	f	59	2	-	2	0	0	0	2	Cryptogenic fibrosis *)
11	m	77	1b	-	3	0	0	0	2	Cryptogenic fibrosis *)

*) underlying etiology remains unknown after extensive clinical, serological, and pathological evaluations.

## Supplementary Materials

The following are available online at https://www.mdpi.com/2073-4409/8/5/512/s1. Supplementary Data 1: Rate equations and kinetic parameter of the kinetic model. Supplementary Data 2: Transfer functions for the relation between plasma hormone concentration and phosphorylation state of interconvertible enzymes, plasma profiles of metabolites and hormones for normal and elevated levels of FFAs. Supplementary Data 3: Example illustrating the imputation method used for the estimation of missing protein intensities. Supplementary Data 4: Measured and imputed protein intensities used for the scaling of v_max_ values of enzymes, transporters and RSPs of the tumors, and the related controls. Supplementary Data 5: Regulatory profiles of tumors and the related controls; TAG content at 10% change of maximal activities of enzymes, transporters, and RSPs.
