# Supplementary material for: Characterization of Lipid and Lipid Droplet Metabolism in Human HCC
Source: Cells. 2019 May 27;8(5):512. doi: 10.3390/cells8050512 (PMC6562484; doi:10.3390/cells8050512)
Supplement: Supplementary file 1 [file cells-08-00512-s001.zip › Supplementary Data 2 - Transfer Functions and Plasma Profiles.docx]

SUPPLEMENTARY DATA 2 - MODELING THE PLASMA PROFILE OF FREE FATTY ACIDS AND HORMONES

***Insulin and glucagon***

The plasma concentrations of the two hormones insulin and glucagon determine the phosphorylation state of the inter-convertible enzymes. Both hormones are secreted by the pancreas into the portal vein and the secretion rate is mainly controlled by the glucose concentration of the blood. Therefore we used the empirical glucose hormone transfer function (GHT), which describes the relationship between the plasma level of glucose and the plasma levels of insulin and glucagon previously established in [[1](#_ENREF_1)].


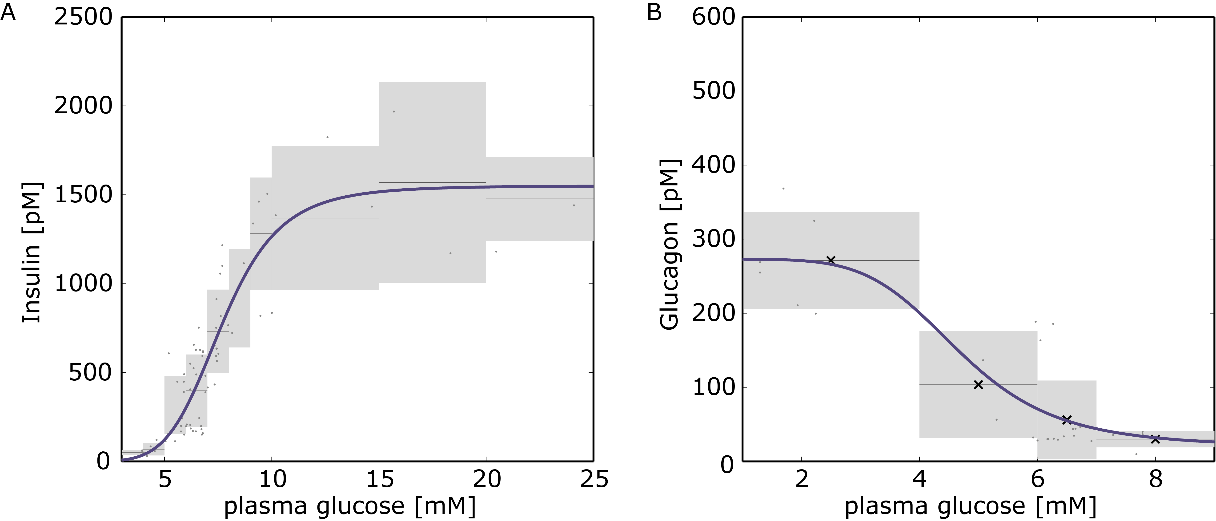


Fig. S1: The GHT functions describe the dependence of plasma insulin (A) and plasma glucagon (B) on plasma glucose levels. Experimentally determined plasma concentrations of glucose and hormone (grey dots) from various sources (insulin: [[2-4](#_ENREF_2)], glucagon: [[5-9](#_ENREF_5)]) were pooled (black lines – mean values, light grey boxes – standard deviations). Periportal hormone concentrations are twice as high as the measured blood plasma concentrations [[5](#_ENREF_5)]. A Hill-type function was used to fit the data by least-square minimization yielding the GHT function.

$$Ins=2*\left( 1.55 nM*\frac{\left( \mathrm{Gl}c_{\mathrm{ext}} \right)^{5.7}}{\left( \mathrm{Gl}c_{\mathrm{ext}} \right)^{5.7}+\left( 7.7 mM \right)^{5.7}} \right)$$

$$Glucagon=2*\left( 0.253 nM*\left( 1-\frac{\left( \mathrm{Gl}c_{\mathrm{ext}} \right)^{5.65}}{\left( \mathrm{Gl}c_{\mathrm{ext}} \right)^{5.65}+\left( 4.7 mM \right)^{5.65}} \right)+0.02 nM \right)$$

The concentration of the hormones determines the phosphorylation state of the interconvertible enzymes [[1](#_ENREF_1)]


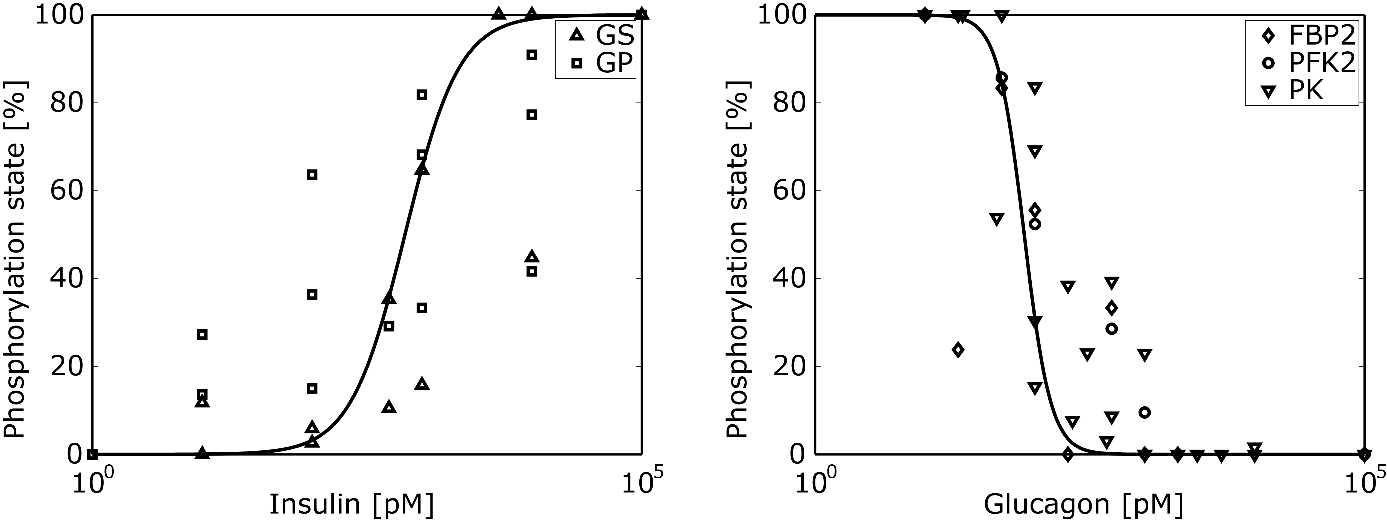


Fig. S2: Hormone phosphorylation function γ. Bold lines depict the function γ used to relate the level of insulin and glucagon to the phosphorylated form of enzymes regulated by reversible phosphorylation. Experimental data are from various sources [[10-17](#_ENREF_10)].

$$\gamma=\frac{1}{2}*\left( 1-\frac{Ins^{1.75}}{Ins^{1.75}+\left( 0.70 nM \right)^{1.75}}+\frac{Glucagon^{3.6}}{Glucagon^{3.6}+\left( 0.08 nM \right)^{3.6}} \right)$$

***Free (non-esterified) fatty acids (FFAs)***

The plasma concentration of FFAs is largely determined by the rate of triglyceride lipolysis in the adipose tissue, which is mainly controlled by insulin and glucagon through the activity of ATGL and the hormone sensitive lipases (HSL). Based on measured relations between the plasma levels of plasma and FFA we constructed an empirical glucose-FFA transfer function (GFT) (see Fig. S3).


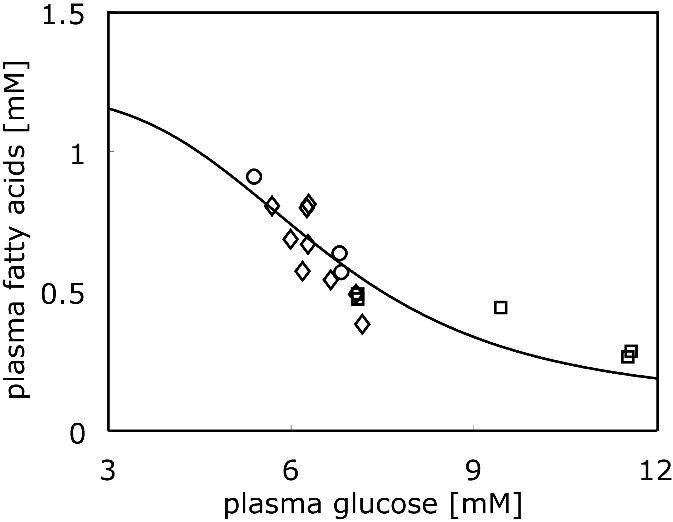


Fig. S3: Glucose-free fatty acids (FFA) transfer (GFT) functions for the dependence of plasma FFA levels from plasma glucose level. Experimentally determined plasma concentration values of glucose and FFAs from various sources: [[18-20](#_ENREF_18)]**.** Hill-type transfer functions were fitted to the data by least-square minimization yielding the GFT.

$$tfa_{plasma}=1.2mM-1.1mM\frac{Glc_{ext}^{4}}{Glc_{ext}^{4}+(6.5m{M)}^{4}}$$

***Fatty acid albumin binding***

Plasma fatty acids are largely bound to plasma albumin and only FFAs are taken up by the liver. We calculated the FFA concentration assuming equilibrium between FFAs and albumin-bound fatty acids. The binding of fatty acids to albumin were described by the following set of differential equations.

The model albumin has five different binding sites for fatty acids that can each be occupied independent of the occupation states of the other sites. Albumin has therefore 32 different occupation states. We denote the occupation state as a binary vector (0 – not occupied; 1 – occupied) of length 5.

Therefore, there are five different reversible elementary processes with rate equations:

$$v_{alb_{txxxx}}=V_{max}^{albumin}\cdot\left( alb_{1xxxx}-\frac{1}{K_{eq}^{alb1}}\cdot c{16}_{ext}\cdot alb_{0xxxx} \right)$$

$$v_{alb_{xt xxx}}=V_{max}^{albumin}\cdot\left( alb_{x1xxx}-\frac{1}{K_{eq}^{alb2}}\cdot c{16}_{ext}\cdot alb_{x0xxx} \right)$$

$$v_{alb_{xxtxx}}=V_{max}^{albumin}\cdot\left( alb_{xx1xx}-\frac{1}{K_{eq}^{alb3}}\cdot c{16}_{ext}\cdot alb_{xx0xx} \right)$$

$$v_{alb_{xxxtx}}=V_{max}^{albumin}\cdot\left( alb_{xxx1x}-\frac{1}{K_{eq}^{alb4}}\cdot c{16}_{ext}\cdot alb_{xxx0x} \right)$$

$$v_{alb_{xxxxt}}=V_{max}^{albumin}\cdot\left( alb_{xxxx1}-\frac{1}{K_{eq}^{alb5}}\cdot c{16}_{ext}\cdot alb_{xxxx0} \right)$$

$K_{eq}^{alb1}=6.2\cdot{10}^{4}$ [[21](#_ENREF_21)]

$K_{eq}^{alb2}=2.3\cdot{10}^{4}$ [[21](#_ENREF_21)]

$K_{eq}^{alb3}=1.2\cdot{10}^{4}$ [[21](#_ENREF_21)]

$K_{eq}^{alb4}=3.1\cdot{10}^{3}$ [[21](#_ENREF_21)]

$K_{eq}^{alb5}=1.5\cdot{10}^{3}$ [[21](#_ENREF_21)]

The t denotes the position where a fatty acid is bound or released. The x denotes either bound or unbound fatty acid at this position. Each elementary process represents 16 actual binding processes.

$$v_{c{16}_{ext}}=\sum{v_{alb}}_{xxxxx}$$

Varying the external fatty acids between 0 and 1.2 mM and calculating the equilibrium FFA concentration results in Fig. S4. We use a fourth order polynomial fit function to calculate the free fatty acids (ffa) from the total fatty acids (tfa) in the plasma (r = 1.0).

$$\frac{ffa_{plasma}}{[nM]}=27.86\left( \frac{tfa_{plasma}}{\left[ mM \right]} \right)^{4}-18.29\left( \frac{tfa_{plasma}}{\left[ mM \right]} \right)^{3}+30.88\left( \frac{tfa_{plasma}}{\left[ mM \right]} \right)^{2}+17.83\left( \frac{tfa_{plasma}}{\left[ mM \right]} \right)$$

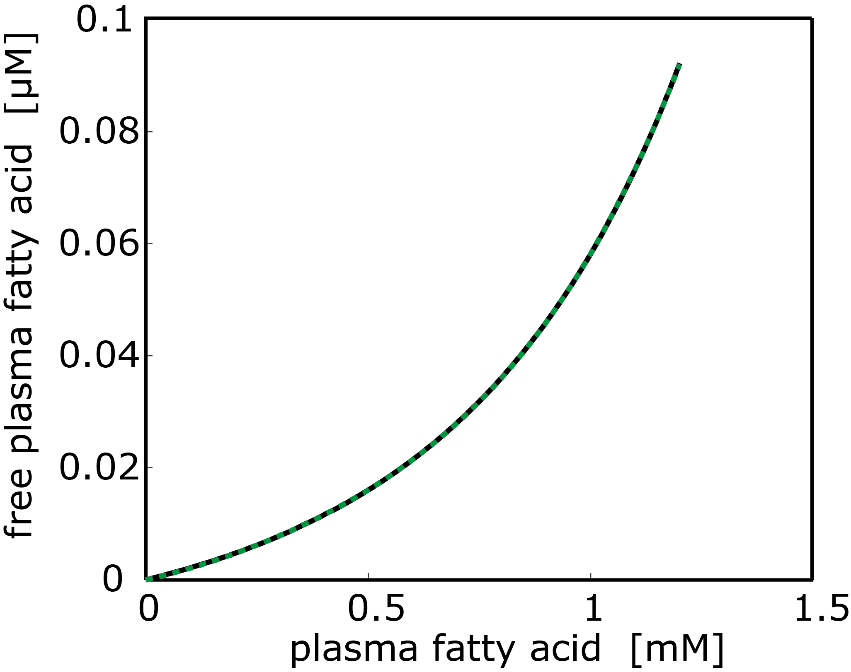


Fig. S4: A plasma albumin concentration of 0.5 mM was used according to [[22](#_ENREF_22)].

***Diurnal plasma metabolite concentrations***


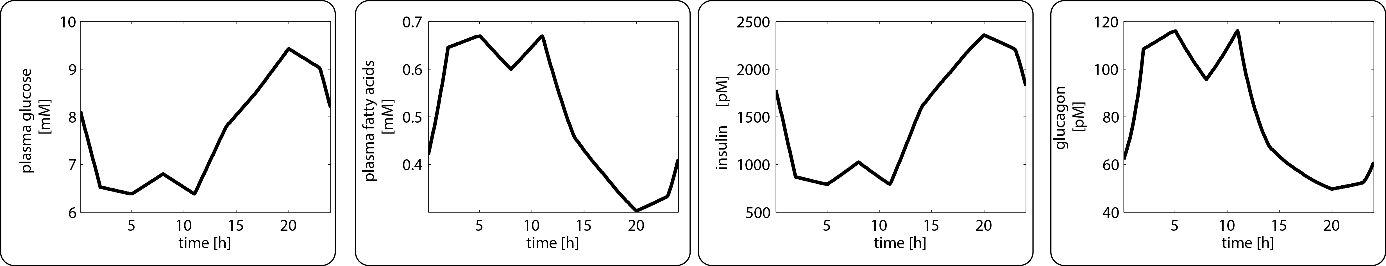


Fig S5: Diurnal plasma profile of glucose, free fatty acids, insulin and glucagon. For the construction of the plasma profile see [[23](#_ENREF_23)].

Table S1: Values normal and high fat conditions.

| **Metabolite** | **Normal condition** | **High fat conditions** |
| --- | --- | --- |
| glucose [mM] | 7.637 | 3.3 |
| fatty acids [mM] | 0.48 | 1.13 |
| Insulin [pM] | 1513 | 24.5 |
| Glucagon [pM] | 70.6 | 485.5 |

References

1. Bulik S, Holzhutter HG, Berndt N: **The relative importance of kinetic mechanisms and variable enzyme abundances for the regulation of hepatic glucose metabolism--insights from mathematical modeling**. *BMC biology* 2016, **14**:15.

2. la Fleur SE, Kalsbeek A, Wortel J, Fekkes ML, Buijs RM: **A daily rhythm in glucose tolerance: a role for the suprachiasmatic nucleus**. *Diabetes* 2001, **50**(6):1237-1243.

3. Frangioudakis G, Gyte AC, Loxham SJ, Poucher SM: **The intravenous glucose tolerance test in cannulated Wistar rats: a robust method for the in vivo assessment of glucose-stimulated insulin secretion**. *J Pharmacol Toxicol Methods* 2008, **57**(2):106-113.

4. Hara E, Saito M: **Diurnal changes in plasma glucose and insulin responses to oral glucose load in rats**. *Am J Physiol* 1980, **238**(5):E463-466.

5. Balks HJ, Jungermann K: **Regulation of peripheral insulin/glucagon levels by rat liver**. *Eur J Biochem* 1984, **141**(3):645-650.

6. Diaz B, Blazquez E: **Effect of pinealectomy on plasma glucose, insulin and glucagon levels in the rat**. *Horm Metab Res* 1986, **18**(4):225-229.

7. Patel DG: **Lack of glucagon response to hypoglycemia in long-term experimental diabetic rats**. *Diabetes* 1983, **32**(1):55-60.

8. Wan CK, Giacca A, Matsuhisa M, El-Bahrani B, Lam L, Rodgers C, Shi ZQ: **Increased responses of glucagon and glucose production to hypoglycemia with intraperitoneal versus subcutaneous insulin treatment**. *Metabolism* 2000, **49**(8):984-989.

9. Zhou H, Tran PO, Yang S, Zhang T, LeRoy E, Oseid E, Robertson RP: **Regulation of alpha-cell function by the beta-cell during hypoglycemia in Wistar rats: the "switch-off" hypothesis**. *Diabetes* 2004, **53**(6):1482-1487.

10. Feliu JE, Hue L, Hers HG: **Hormonal control of pyruvate kinase activity and of gluconeogenesis in isolated hepatocytes**. *Proc Natl Acad Sci U S A* 1976, **73**(8):2762-2766.

11. Claus TH, El-Maghrabi MR, Pilkis SJ: **Modulation of the phosphorylation state of rat liver pyruvate kinase by allosteric effectors and insulin**. *J Biol Chem* 1979, **254**(16):7855-7864.

12. Pilkis S, Schlumpf J, Pilkis J, Claus TH: **Regulation of phosphofructokinase activity by glucagon in isolated rat hepatocytes**. *Biochem Biophys Res Commun* 1979, **88**(3):960-967.

13. Schudt C: **Regulation of glycogen synthesis in rat-hepatocyte cultures by glucose, insulin and glucocorticoids**. *Eur J Biochem* 1979, **97**(1):155-160.

14. El-Maghrabi MR, Claus TH, Pilkis J, Fox E, Pilkis SJ: **Regulation of rat liver fructose 2,6-bisphosphatase**. *J Biol Chem* 1982, **257**(13):7603-7607.

15. Bartrons R, Hue L, Van Schaftingen E, Hers HG: **Hormonal control of fructose 2,6-bisphosphate concentration in isolated rat hepatocytes**. *Biochem J* 1983, **214**(3):829-837.

16. Hartmann H, Probst I, Jungermann K, Creutzfeldt W: **Inhibition of glycogenolysis and glycogen phosphorylase by insulin and proinsulin in rat hepatocyte cultures**. *Diabetes* 1987, **36**(5):551-555.

17. Syed NA, Khandelwal RL: **Reciprocal regulation of glycogen phosphorylase and glycogen synthase by insulin involving phosphatidylinositol-3 kinase and protein phosphatase-1 in HepG2 cells**. *Mol Cell Biochem* 2000, **211**(1-2):123-136.

18. De Gasquet P, Griglio S, Pequignot-Planche E, Malewiak MI: **Diurnal changes in plasma and liver lipids and lipoprotein lipase activity in heart and adipose tissue in rats fed a high and low fat diet**. *J Nutr* 1977, **107**(2):199-212.

19. Yamamoto H, Nagai K, Nakagawa H: **Role of SCN in daily rhythms of plasma glucose, FFA, insulin and glucagon**. *Chronobiol Int* 1987, **4**(4):483-491.

20. Djordjevic J, Jasnic N, Vujovic P, Djurasevic S, Djordjevic I, Cvijic G: **The Effect of Fasting on the Diurnal Rhythm of Rat Acth and Corticosterone Secretion**. *Archives of Biological Sciences* 2008, **60**(4):541-546.

21. Spector AA: **Fatty-Acid Binding to Plasma Albumin**. *Journal of Lipid Research* 1975, **16**(3):165-179.

22. Rose R, Klemcke HG: **Relationship between Plasma Albumin Concentration and Plasma Volume in 5 Inbred Rat Strains**. *Journal of the American Association for Laboratory Animal Science* 2015, **54**(5):459-464.

23. Berndt N, Bulik S, Wallach I, Wunsch T, Konig M, Stockmann M, Meierhofer D, Holzhutter HG: **HEPATOKIN1 is a biochemistry-based model of liver metabolism for applications in medicine and pharmacology**. *Nature communications* 2018, **9**(1):2386.
