# Supplementary material for: Characterization of Lipid and Lipid Droplet Metabolism in Human HCC
Source: Cells. 2019 May 27;8(5):512. doi: 10.3390/cells8050512 (PMC6562484; doi:10.3390/cells8050512)
Supplement: Supplementary file 1 [file cells-08-00512-s001.zip › Supplementary Data 3 - Illustration of the Imputation Method.docx]

Supplementary DatA 3 - Example illustrating the imputation method used for the estimation of missing protein intensities

First, we compiled a list of potential predictor proteins for which measured intensities were available for all eleven controls and eleven tumors. This resulted in a set of 1,675 predictor proteins. Second, for a given target protein with missing protein intensity values in some tumors, we determined a group of predictor proteins exhibiting a Spearman’s correlation larger than a critical threshold value R_crit_ = 0.8. Linear regression analysis with each of these predictor proteins yielded estimates of the missing values. Finally, the missing intensity values were filled by the mean of the estimates obtained for all predictor proteins. The quality of the imputation was assessed by the coefficient of variation (standard deviation devised by the mean). We included in the imputation procedure only target proteins comprising at least four out of eleven experimentally determined protein intensities as otherwise the chance of randomly high correlations was too large. An example illustrating the used imputation method is shown here. The list of measured and imputed protein intensities used for model calibration is given in Supplementary Data 4.


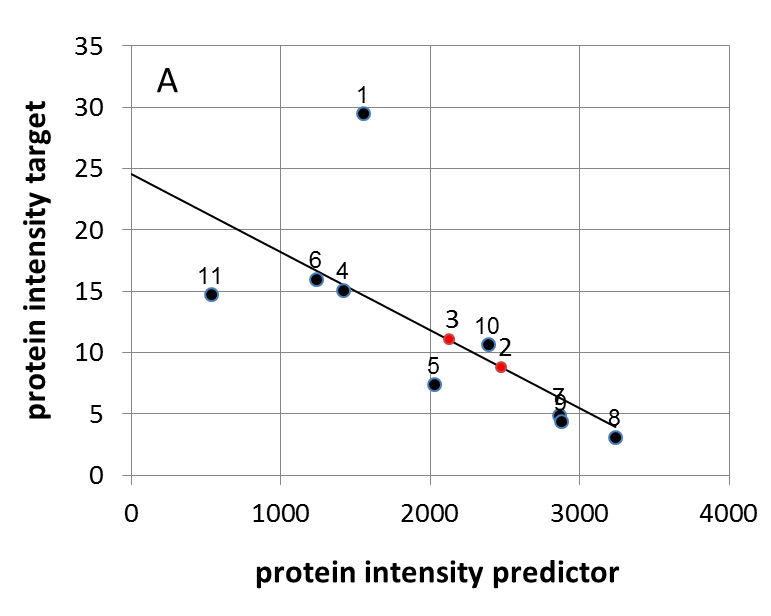

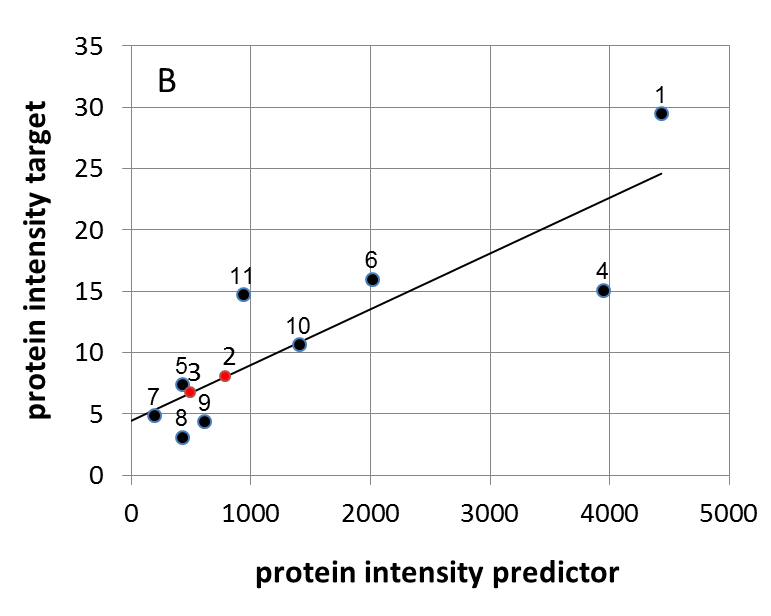


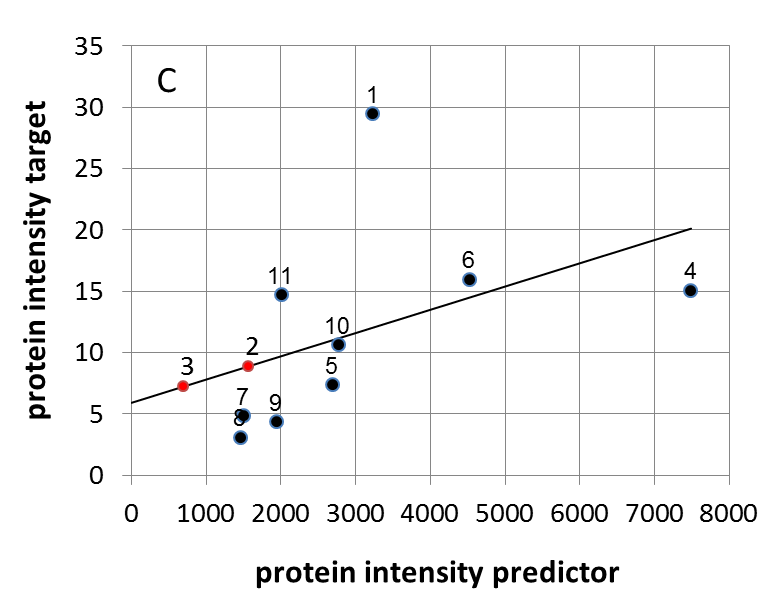


Fig. S6: Example for the imputation of missing protein intensities for model protein #11 (Monoacylglycerol lipase, UniProtID: Q8N2K0) in HCC#2 and HCC#3. Protein intensities are given as 10^-6^ MaxQuant peak area. In this example, the missing intensities for model protein #11 in HCC#2 and HCC#3 are inferred by linear regression (red dots) with respect to three predictor proteins, which correlate with the experimentally determined protein intensities of protein #11 (black dots) with a Spearman’s correlation coefficient larger than 0.8.
A) Predictor protein: Histone H3 (UniProtID: P84243). Estimated protein intensities: 8.8 (T2); 11.0 (T3).
B) Predictor protein: Transthyretin precursor (UniProtID: A0A087WV45). Estimated protein intensities: 8.1 (T2); 6.7 (T3).
C) Predictor protein: Cofilin-1 (UniProtID: E9PP50). Estimated protein intensities: 8.9 (T2); 7.2 (T3).
Imputed value T2: estimated mean = 8.6; SD = 0.36; CV = 0.041
Imputed value T3: estimated mean = 8.3; SD = 1.92; CV = 0.23
