# Supplementary material for: Characterization of Lipid and Lipid Droplet Metabolism in Human HCC
Source: Cells. 2019 May 27;8(5):512. doi: 10.3390/cells8050512 (PMC6562484; doi:10.3390/cells8050512)
Supplement: Supplementary file 1 [file cells-08-00512-s001.zip › Supplementary Data 1 - Model Documentation.docx]

Supplementary Data 1 – MODEL DOCUMENTATION

Free fatty acid uptake by a combination of diffusion and an active transport process with CD36

$$FA_{ex}\leftrightarrow FA_{cyt}$$

$$v_{diff}=V_{max}^{diff}\cdot({FA}_{ex}-FA_{cyt})$$

$$v_{CD36}= V_{max}^{CD36}\frac{\left( {FA}_{ex}-FA_{cyt} \right)}{\left( 1+\frac{{FA}_{ex}}{K_{m}^{FA_{ex}}}+\frac{FA_{cyt}}{K_{m}^{FA_{cyt}}} \right)}$$

$K_{m}^{FA_{ex}}=0.000083 mM$ [1] $\{Kampf, 2004 \#20\}$

$K_{m}^{FA_{cyt}}=0.004 mM$ [2]

Activation of free fatty acids by Acyl-CoA Synthetase

$$FA_{cyt}+\mathrm{CoA}_{\mathrm{cyt}}+ATP\leftrightarrow FA_{CoA}+ADP$$

$$v_{ACSL1}=V_{max}^{ACSL1}\cdot\frac{c{16}_{cyt}}{c{16}_{cyt}+K_{m}^{c{16}_{cyt}}}\cdot\frac{atp_{cyt}}{atp_{cyt}+K_{m}^{atp_{cyt}}}\cdot\frac{coa_{cyt}}{coa_{cyt}+K_{m}^{coa_{cyt}}}$$

$V_{max}^{ACSL1}$ for numerical value see Supplementary Data 4

$K_{m}^{c{16}_{cyt}}=$ 0.005 [3]

$K_{m}^{atp_{cyt}}=$ 0.649 [3]

$K_{m}^{coa_{cyt}}=$ 0.0064 [3]

$$v_{ACSL4}=V_{max}^{ACSL4}\cdot\frac{c{16}_{cyt}}{c{16}_{cyt}+K_{m}^{c{16}_{cyt}}}\cdot\frac{atp_{cyt}}{atp_{cyt}+K_{m}^{atp_{cyt}}}\cdot\frac{coa_{cyt}}{coa_{cyt}+K_{m}^{coa_{cyt}}}$$

$V_{max}^{ACSL4}$ for numerical value see Supplementary Data 4

$K_{m}^{c{16}_{cyt}}=$ 0.0054 [3]

$K_{m}^{atp_{cyt}}=$ 0.034 [3]

$K_{m}^{coa_{cyt}}=$ 0.0041 [3]

$$v_{ACSL5}=V_{max}^{ACSL5}\cdot\frac{c{16}_{cyt}}{c{16}_{cyt}+K_{m}^{c{16}_{cyt}}}\cdot\frac{atp_{cyt}}{atp_{cyt}+K_{m}^{atp_{cyt}}}\cdot\frac{coa_{cyt}}{coa_{cyt}+K_{m}^{coa_{cyt}}}$$

$V_{max}^{ACSL5}$ for numerical value see Supplementary Data 4

$K_{m}^{c{16}_{cyt}}=$ 0.0086 [3]

$K_{m}^{atp_{cyt}}=$ 0.666 [3]

$K_{m}^{coa_{cyt}}=$ 0.0024 [3]

Mitochondrial β-oxidation by Carnitine palmitoyltransferase 1 (CPT1)

$${FA}_{CoA}+Car_{cyt}{\to FA}_{Car}+{CoA}_{cyt}$$

$$v_{CPT1}=V_{max}^{CPT1}\cdot\frac{{FA}_{CoA}^{n}\cdot Car_{cyt}}{\left( {FA}_{CoA}^{n}+\left( K_{m}^{FA_{CoA}} \right)^{n} \right)\cdot\left( Car_{cyt}+K_{m}^{Car_{cyt}} \right)}$$

$n=2.47$ [4]

$$K_{m}^{FA_{CoA}}=K_{0}^{FA_{CoA}}\cdot\left( 1+\frac{malcoa_{cyt}}{K_{i}^{malcoa_{cyt}}} \right)$$

$K_{0}^{FA_{CoA}}=0.03 \mathrm{mM}$[5]

$K_{i}^{malcoa_{cyt}}=0.0025 \mathrm{mM}$ [6]

$K_{m}^{Car_{cyt}}=0.032 \mathrm{mM}$[7]

Esterification of Glycerol-3-phosphate by glycerol-3-phosphate acyltransferases (GPAT)

$$G3p+FA_{CoA} \underset{\to}{v_{GPAT}}lpa_{er}+CoA_{cyt}$$

$$v_{gpat}=V_{max}^{gpat}\cdot\left( \frac{G3p_{cyt}}{G3p_{cyt}+K_{m}^{G3p_{cyt}}} \right)\cdot\left( \frac{FA_{CoA}}{FA_{CoA}+K_{m}^{FA_{CoA}}} \right)$$

$K_{m}^{G3p_{cyt}}=0.2 mM$ [8]

$K_{m}^{FA_{CoA}}=0.09 mM$[9]

Esterification of lysophosphatidic acid with a long-chain acyl-CoA by acylglycerolphosphate acyltransferase (AGPAT)

$$lpa_{er}+FA_{CoA} \to pa_{er}+CoA_{cyt}$$

$$v_{agpat}=V_{max}^{agpat}\cdot\left( \frac{lpa_{er}}{lpa_{er}+K_{m}^{lpa_{er}}} \right)\cdot\left( \frac{FA_{CoA}}{FA_{CoA}+K_{m}^{FA_{CoA}}} \right)$$

$K_{m}^{lpa_{er}}=0.0065$ mM [10]

$K_{m}^{FA_{CoA}}=0.004 \mathrm{mM}$[10]

Hydrolysis of phosphatidate by phosphatidate phosphatase-1 (PAP)

$$pa_{er}\to DAG_{ER}+{Pi}_{cyt}$$

$$v_{pap}=V_{max}^{pap}\cdot\left( \frac{pa_{er}^{n}}{pa_{er}^{n}+\left( K_{m}^{pa_{er}} \right)^{n}} \right)$$

$K_{m}^{pa_{er}}=0.35$ [11]

$n=2.2$ [11]

Phospholipid synthesis by diacylglycerol choline phosphotransferase (CPT)

$$DAG_{ER} \to PC_{ER}$$

$$v_{CPT}=V_{max}^{CPT}\cdot\left( \frac{DAG_{er}}{DAG_{er}+K_{m}^{DAG_{er}}} \right)$$

$K_{m}^{DAG_{er}}=0.125$ [12]

Phosphatidylcholine export

$$v_{PC_{exp}}= v_{\max}^{\mathrm{PCT}}\cdot{PC}_{ER}$$

Synthesis and degradation of ApoB

$$\underset{\to}{v_{syn}}ApoB\underset{\to}{v_{deg}}$$

Degradation of ApoB is the sum a basal and an insulin dependent degradation rate.

$$v_{ApoB}^{syn}= const.$$

$v_{ApoB}^{deg}=V_{max}^{ApoB-deg}\cdot ApoB\cdot\left( k_{1}+k_{2}\frac{ins_{ext}}{ins_{ext}+K_{m}^{ins_{ext}}} \right)$

$k_{1}=0.3$ [13]

$k_{2}=0.7$ [13]

$K_{m}^{ins_{ext}}={10}^{3}pM$ [13]

Synthesis and Secretion of VLDL

The very low density lipoprotein (VLDL) is synthesized by MTP mediated transfer of TAG from the ER to ApoB.

$$TAG_{ER1} \to TAG_{VLDL}$$

$$v_{MTP}= v_{{max}_{MTP}}\cdot{TAG}_{ER}\cdot apoB$$

$$TAG_{VLDL} \to VLDL_{ex}$$

$$v_{VLDL_{ex}}= v_{{max}_{VLDL_{ex}}}\cdot\frac{TAG_{VLDL}}{TAG_{VLDL}+K_{m}^{TAG_{VLDL}}}$$

$$K_{m}^{TAG_{VLDL}}=10 mM$$

Synthesis of TAG by DGAT1

DGAT1 resides in the ER membrane and synthesizes $TAG_{ER1}$ used for VLDL synthesis and nascent LD formation.

$$DAG_{ER}+FA_{CoA}\to TAG_{ER1}+CoA_{cyt}$$

$$v_{TAG_{ER}}^{DGAT1}=v_{\max}^{DGAT1}\cdot\frac{{FA}_{CoA}}{\left( {FA}_{CoA}+K_{m}^{FFA_{CoA}} \right)}\frac{DAG_{ER}}{\left( DAG_{ER}+K_{m}^{DAG_{er}} \right)}$$

$K_{m}^{DAG}=0.03 mM$ [14]

$K_{m}^{FFA_{CoA}}=0.1 mM$ [15]

Synthesis of TAG and filling of LDs by DGAT2

DGAT2 resides either in the ER membrane and synthesizes $TAG_{ER2}$ used nascent LD formation or distributes to LD surface synthesizing$TAG_{LD^{n}}$, directly filling LD of class n.

$$v_{TAG_{ER2}}^{DGAT2}=v_{\max_{\mathrm{ER}}}^{DGAT2}\cdot\frac{{FA}_{CoA}}{\left( {FA}_{CoA}+K_{m}^{FA_{CoA}} \right)}\frac{DAG_{ER}}{\left( DAG_{ER}+K_{m}^{DAG_{er}} \right)}$$

$$v_{{TAG}_{LD^{n}}}^{DAGT2}=v_{\max_{\mathrm{LD}^{n}}}^{DGAT2}\cdot\frac{{FA}_{CoA}}{\left( {FA}_{CoA}+K_{m}^{FFA_{CoA}} \right)}\frac{DAG_{ER}}{\left( DAG_{ER}+K_{m}^{DAG_{er}} \right)}$$

$K_{m}^{DAG}=0.03 mM$ [14]

$K_{m}^{FA_{CoA}}=0.1 mM$ [15]

The distribution of DGAT2 between the ER and the different LD classes $LD^{n}$ depends on the number of LDs in each fraction $num_{LD^{n}}$ and the radius $r_{ld}$ of the LDs.

$$K_{LD^{n}}^{dgat2}=num_{LD^{n}}\cdot\left( 1-\frac{r_{ld}^{n}}{r_{ld}^{n}+k_{r_{ld}}^{n}} \right)$$

$$n=3$$

$$k_{r_{ld}}^{n}=0.15 \mu m$$

$$K_{er}^{dgat2}=\frac{10}{\sum_{n=1}^{LD_{max}} num_{LD^{n}}}$$

$$v_{\max_{\mathrm{ER}}}^{DGAT2}=v_{max}^{DGAT2}\cdot\frac{K_{er}^{dgat2}}{K_{er}^{dgat2}+\sum_{n=1}^{LD_{max}} K_{LD^{n}}^{dgat2}}$$

$$v_{\max_{\mathrm{LD}^{n}}}^{DGAT2}= v_{max}^{DGAT2}\cdot\frac{K_{LD^{n}}^{dgat2}}{K_{er}^{dgat2}+\sum_{n=1}^{LD_{max}} K_{LD^{n}}^{dgat2}}$$

Filling of LDs of class n diminishes the cellular content $TAG_{LD^{n}}$ in the respective class and increases the cellular content of $TAG_{LD^{n+1}}$in class n+1 by shifting TAG from $TAG_{LD^{n}}$ to $TAG_{LD^{n+1}}$. This shifting rate depends on the relative volume $\Delta Vol_{LD^{n}}$ between class n and class n+1.

$$v_{shift_{LD^{n}}}^{DGAT2}=v_{{TAG}_{LD^{n-1}}}^{DAGT2}\cdot\Delta Vol_{LD^{n}}$$

$$\Delta Vol_{LD^{n}}=\frac{Vol_{LD^{n}}}{Vol_{LD^{n-1}}}-1$$

The change of $TAG_{LD^{n}}$ is the sum of filling of this class accompanied by shifting of $TAG_{LD^{n-1}}$ into this class, and shifting from this class in the next bigger class.

$$v_{TAG_{LD^{n}}}^{fill_{DGAT2}}=v_{{TAG}_{LD^{n}}-1}^{DAGT2}+ v_{shift_{LD^{n}}}^{DGAT2}- v_{shift_{LD^{n+1}}}^{DGAT2}$$

Synthesis of nascent Lipid Droplet (without protein coating)

Nascent lipid droplets, $TAG_{nLD}$,lacking protein coating are synthesized from the ER membrane TAG pools.

$$TAG_{ER1/2}\to LD_{nLD}$$

$$v_{{LD}_{1}}^{de novo}=v_{{max}_{LD1}}^{de novo}\cdot{tag}_{ER1}$$

$$v_{{LD}_{2}}^{de novo}=v_{{max}_{LD2}}^{de novo}\cdot{tag}_{ER2}$$

Perilipin (Plin1)

$$\underset{\to}{v_{on}^{Plin1}}{Plin1}_{LD}\underset{\to}{v_{off}^{Plin1}}$$

Plin1 binds reversibly to large LD in dependence on LD radius and fraction of free LD surface $f_{LD}^{free}$ . ${Plin1}_{LD}$ describes the fraction of LD surface occupied by $Plin1$.

$$v_{on}^{Plin1}=k_{on}^{Plin1}\cdot k_{r}^{Plin1}\cdot{Plin1}_{cyt}\cdot f_{LD}^{free}$$

$$v_{off}^{Plin1}=k_{off}^{Plin1}\cdot(1-k_{r}^{Plin1})\cdot Plin1_{LD}$$

$$k_{r}^{Plin1}=\frac{r_{LD}^{n}}{r_{LD}^{n}+k_{r_{ld}}^{n}}$$

$$k_{r_{ld}}=2 \mu m$$

$$n=15$$

ADRP (Perilipin 2)

$$\underset{\to}{v_{on}^{ADRP}}{ADRP}_{LD}\underset{\to}{v_{off}^{ADRP}}$$

ADRP binds reversibly to medium size LD in dependence on LD radius and fraction of free LD surface $f_{LD}^{free}$. ${ADRP}_{LD}$ describes the fraction of LD surface occupied by $ADRP$.

$$v_{on}^{ADRP}={k_{on}^{ADRP}\cdot(1-k_{r}^{ADRP})\cdot ADRP}_{cyt}\cdot f_{LD}^{free}$$

$$v_{off}^{ADRP}=k_{off}^{ADRP}\cdot ADRP_{LD}$$

$$k_{r}^{ADRP}=\frac{r_{LD}^{n}}{r_{LD}^{n}+k_{r_{ld}}^{n}}$$

$$k_{r_{ld}}=2 \mu m$$

$$n=12$$

Tip47 (Perilipin 3)

$$\underset{\to}{v_{on}^{TIP47}}{TIP47}_{LD}\underset{\to}{v_{off}^{TIP47}}$$

Tip47 reversibly binds primary to small lipid droplets in dependence on LD radius and fraction of free LD. ${TIP47}_{LD}$ describes the fraction of LD surface occupied by $TIP47$.

$$v_{on}^{TIP47}={k_{on}^{TIP47}\cdot TIP47}_{cyt}\cdot f_{LD}^{free}\cdot k_{r}^{TIP47}$$

$$v_{off}^{TIP47}=k_{off}^{TIP47}\cdot(1-k_{r}^{TIP47})\cdot TIP{47}_{LD}$$

$$k_{r}^{TIP47}=\left( 1-\frac{r_{LD}^{n}}{r_{LD}^{n}+k_{r_{ld}}^{n}} \right)$$

$$k_{r_{ld}}=0.5 \mu m$$

$$n=10$$

Nascent Lipid Droplet coating with Tip47

Nascent lipid droplets are coated with Tip47.

$$TAG_{nld}+Tip{47}_{cyt}\to TAG_{L}$$

$$v_{LD_{1}}^{TIP47}={k_{cat}\cdot TAG}_{nLD}\cdot Tip{47}_{cyt}$$

Binding of CGI58 to LD

$$\underset{\to}{v_{on}^{CGI58}}{CGI58}_{LD}\underset{\to}{v_{off}^{CGI58}}$$

$$v_{on}^{CGI58}=k_{on}^{CGI58}\cdot{CGI58}_{cyt}\cdot{(k_{1}\cdot Pln1}_{LD}+k_{2}\cdot ADRP_{LD})$$

$$v_{off}^{CGI58}={k_{off}^{CGI58}\cdot CGI58}_{LD}$$

$$k_{1}=0.1$$

$$k_{2}=0.2$$

Binding of ATGL to LD

ATGL binds to fraction of empty LD surface ($f_{LD}^{free}$). Phosphorylation ($\gamma$) of CGI58 in response to hormonal stimulation leads to binding to and thereby activation of ATGL.

$$v_{{ATGL}_{LD}}^{on}=k_{{ATGL}_{LD}}^{on}\cdot{num}_{LD}\cdot{Sur}_{LD}\cdot f_{LD}^{free}\cdot{ATGL}_{cyt}$$

$$v_{{ATGL}_{LD}}^{off}=k_{{ATGL}_{LD}}^{off}\cdot{ATGL}_{LD}$$

$$v_{{ATGL}_{LD}}^{CGI58 on}=k_{{ATGL}_{LD}}^{CGI58 on}\cdot{num}_{LD}\cdot{Sur}_{LD}\cdot({CGI58}_{LD}\cdot\left( 1-\gamma\right))\cdot{ATGL}_{LD}$$

$$v_{{ATGL}_{LD}}^{CGI58 off}=k_{{ATGL}_{LD}}^{CGI58 off}\cdot{ATGL}_{LD}^{CGI58}$$

Lipid droplet degradation by adipose triglyceride lipase (ATGL)

$$TAG_{LD} \to DAG_{LD}+FA_{cyt}$$

The first step in LD triglyceride degradation is performed by ATGL.

$$v_{ATGL_{LD}}= v_{max}^{ATGL}\cdot{num}_{LD}\cdot({ATGL}_{LD}+20\cdot{ATGL}_{LD}^{CGI58})\cdot{TAG}_{LD}$$

$$v_{ATGL}^{TAG^{nld}}={k_{cat}\cdot TAG}_{nld}\cdot{ATGL}_{cyt}$$

Lipid droplet degradation by hormone sensitive lipase (HSL)

$$TAG_{LD} \to DAG_{LD}+FA_{cyt}$$

$$DAG_{LD} \to MAG_{LD}+FA_{cyt}$$

HSL can hydrolyze TAG, although its main activity is DAG hydrolyzation. HSL is activated by phosphorylation of $ADRP_{LD}+Plin1_{LD}$.

$$v_{HSL}^{TAG}= v_{\max_{\mathrm{TAG}}}^{HSL}\cdot{\left( 1-\gamma\right)\cdot num}_{LD}\cdot{Sur}_{LD}\cdot(f_{LD}^{free} + ADRP_{LD}+Plin1_{LD})\cdot\frac{{TAG}_{LD}}{{TAG}_{LD}+{DAG}_{LD}+{MAG}_{LD}}$$

$$v_{HSL}^{DAG}= v_{{max}_{DAG}}^{HSL}\cdot(1-\gamma)\cdot{num}_{LD}\cdot{Sur}_{LD}\cdot(f_{LD}^{free} +ADRP_{LD}+Plin1_{LD})\cdot\frac{{DAG}_{LD}}{{TAG}_{LD}+{DAG}_{LD}+{MAG}_{LD}}$$

Hydrolysis of monoacylglycerol by monoacylglycerol lipase (MGL)

$$MAG_{LD} \to FA_{cyt}$$

Final step in LD triglyceride degradation is performed by MGL.

$$v_{MGL}= v_{{max}_{MAG}}^{MGL}\cdot{num}_{LD}\cdot{Sur}_{LD}\frac{{MAG}_{LD}}{({TAG}_{LD}+{DAG}_{LD}+{MAG}_{LD})}$$

Lipid droplet fusion

The fusion of lipid droplets is regulated by fsp27. The fusion rate $fr_{ij}$ between LDs of size i and j depends on the radii of the two LDs involved: The larger the size difference the faster the fusion rate. LDs with maximal size ($\max_{LD}$) cannot fuse.

$$fr_{ij}=V_{max}^{fus}\cdot{fsp27\cdot num}_{LD^{i}}\cdot{num}_{LD^{j}}\cdot\left( \left| \frac{1}{r_{LD^{j}}}-\frac{1}{r_{LD^{i}}} \right| \right)$$

Fusion of LDs of class i and j with i>j diminishes the cellular content $TAG_{LD^{i}}$and $TAG_{LD^{j}}$ in the respective class and increases the cellular content of $TAG_{LD^{j+1}}$. $TAG_{LD^{j}}$is filled with $TAG_{LD^{i}}$. Analogous to LD filling, TAG is shifted from $TAG_{LD^{j}}$ to $TAG_{LD^{j+1}}$. This shifting rate depends on the relative volume $\Delta Vol_{LD^{j}}$ between class j and class j+1.

$$v_{{TAG}_{LD^{j}}}^{fus_{shrink}}=\sum_{i=j}^{\max_{LD} -1} fr_{ij}\cdot Vol_{LD^{j}}$$

$$v_{shift_{LD^{j}}}^{fus_{shrink}}=v_{{TAG}_{LD^{j}}}^{fus_{shrink}}\cdot\Delta Vol_{LD^{j}}$$

$$v_{{TAG}_{LD^{j}}}^{fus_{growth}}=\sum_{i=1}^{j-1} fr_{ij}\cdot Vol_{LD^{i}}$$

$$v_{shift_{LD^{j}}}^{fus_{growth}}=v_{{TAG}_{LD^{j}}}^{fus_{growth}}\cdot\Delta Vol_{LD^{j}}$$

$$v_{Tag_{LD^{j}}}^{fus}=v_{{TAG}_{LD^{j}}}^{fus_{growth}}-v_{{TAG}_{LD^{j}}}^{fus_{shrink}}+v_{shift_{LD^{j}}}^{fus}-v_{shift_{LD^{j+1}}}^{fus}$$

$${Vol}_{cyt}=2.81*{10}^{-12}L$$

$${Vol}_{ER}=2.1*{10}^{-12}L$$

$${Vol}_{VLDL}= {Vol}_{cyt}$$

$${Vol}_{LD}= {Vol}_{cyt}$$

Stoichiometric matrix

$\frac{d {FA}_{cyt}}{dt}=v_{CD36}{+ v}_{diff}-v_{ACS}+\sum v_{HSL}^{TAG}+\sum v_{ATGL_{LD}}+\sum v_{HSL}^{DAG}+\sum v_{MGL}$

$$\frac{d {FA}_{CoA}}{dt}=v_{ACS}-v_{gpat}\cdot\frac{{Vol}_{ER}}{{Vol}_{cyt}}-v_{agpat}\cdot\frac{{Vol}_{ER}}{{Vol}_{cyt}}-v_{CPT1}-\left( \sum v_{{TAG}_{LD^{n}}}^{DAGT2} \right)-v_{TAG_{ER2}}^{DGAT2}\cdot\frac{{Vol}_{ER}}{{Vol}_{cyt}}-v_{TAG_{ER}}^{DGAT1}\cdot\frac{{Vol}_{ER}}{{Vol}_{cyt}}$$

$$\frac{d {Co}_{cyt}}{dt}=v_{gpat}\cdot\frac{{Vol}_{ER}}{{Vol}_{cyt}}+v_{agpat}\cdot\frac{{Vol}_{ER}}{{Vol}_{cyt}}-v_{ACS}+v_{CPT1}+\left( \sum v_{{TAG}_{LD^{n}}}^{DAGT2} \right)+v_{TAG_{ER2}}^{DGAT2}\cdot\frac{{Vol}_{ER}}{{Vol}_{cyt}}+v_{TAG_{ER}}^{DGAT1}\cdot\frac{{Vol}_{ER}}{{Vol}_{cyt}}$$

$$\frac{d {PC}_{ER}}{dt}=v_{CPT}-v_{PC_{exp}}$$

$$\frac{d LPA}{dt}=v_{gpat}-v_{agpat}$$

$$\frac{d PA}{dt}={-v}_{pap}+v_{agpat}$$

$$\frac{d {MAG}_{cyt}}{dt}=\sum v_{HSL}^{DAG}-v_{MGL}$$

$$\frac{d {DAG}_{ER}}{dt}=v_{pap}-v_{CPT}-\left( \sum v_{{TAG}_{LD^{n}}}^{DAGT2} \right)\cdot\frac{{Vol}_{ER}}{{Vol}_{cell}}-v_{TAG_{ER2}}^{DGAT2}-v_{TAG_{ER}}^{DGAT1}$$

$$\frac{d {DAG}_{LD}}{dt}=v_{ATGL_{LD}}+v_{HSL}^{TAG}-v_{HSL}^{DAG}$$

$$\frac{d {MAG}_{LD}}{dt}=v_{HSL}^{DAG}-v_{MGL}$$

$$\frac{d {TAG}_{ER2}}{dt}=v_{TAG_{ER2}}^{DGAT2}-\sum\left( v_{{LD}_{2}}^{de novo}\cdot\frac{{Vol}_{ER}}{{Vol}_{cyt}} \right)$$

$$\frac{d {TAG}_{ER1}}{dt}=-v_{MTP}-\sum\left( v_{{LD}_{1}}^{de novo}\cdot\frac{{Vol}_{ER}}{{Vol}_{cyt}} \right)+v_{TAG_{ER}}^{DGAT1}$$

$$\frac{d {TAG}_{LD}}{dt}=v_{TAG_{LD^{n}}}^{fill_{DGAT2}}-v_{ATGL}-v_{HSL}^{TAG}+v_{Tag_{LD^{n}}}^{fus}$$

$$\frac{d {TAG}_{nld}}{dt}=v_{{LD}_{1}}^{de novo}+v_{{LD}_{2}}^{de novo}-v_{LD_{1}}^{TIP47}-v_{ATGL}^{TAG^{nld}}$$

$$\frac{d {TAG}_{VLDL}}{dt}={v_{MTP}\cdot\frac{{Vol}_{ER}}{{Vol}_{VLDL}}-v}_{VLDL_{ex}}$$

$$\frac{d ApoB}{dt}=v_{ApoB}^{syn}-v_{ApoB}^{deg}-v_{MTP}$$

$$\frac{d{Plin1}_{LD}}{dt}=v_{on}^{Plin1}-v_{off}^{Plin1}$$

$$\frac{dADRP_{LD}}{dt}=v_{on}^{ADRP}-v_{off}^{ADRP}$$

$$\frac{d{TIP47}_{LD}}{dt}=v_{on}^{TIP47}-v_{off}^{TIP47}$$

$$\frac{d{CGI58}_{LD}}{dt}=v_{on}^{CGI58}-v_{off}^{CGI58}-v_{{ATGL}_{LD}}^{CGI58 on}+v_{{ATGL}_{LD}}^{CGI58 off}$$

$$\frac{d{CGI58}_{LD}^{ATGL}}{dt}=v_{{ATGL}_{LD}}^{CGI58 on}-v_{{ATGL}_{LD}}^{CGI58 off}$$

$$\frac{d{ATGL}_{LD}}{dt}=v_{{ATGL}_{LD}}^{on}-v_{{ATGL}_{LD}}^{off}-v_{{ATGL}_{LD}}^{CGI58 on}+v_{{ATGL}_{LD}}^{CGI58 off}$$

$$\frac{d{ATGL}_{LD}^{CGI58}}{dt}=v_{{ATGL}_{LD}}^{CGI58 on}-v_{{ATGL}_{LD}}^{CGI58 off}$$

$$\frac{df_{LD}^{free}}{dt}=-\frac{d{Plin1}_{LD}}{dt}-\frac{dADRP_{LD}}{dt}-\frac{d{TIP47}_{LD}}{dt}-\frac{d{ATGL}_{LD}}{dt}$$

List of the maximal enzyme activities

| Identifier | Name | Maximal activity (Vmax-value/k_cat_) |
| --- | --- | --- |
| $v_{diff}$ | Diffusive FA uptake | 1.2442e+05 h^-1^ |
| $v_{CD36}$ | CD36 dependent FA uptake | 9.3312e+05 h^-1^ |
| $v_{ACS}$ | Acyl-Coa Synthase | 3.1104e+06 mM/h |
| $v_{CPT1}$ | Carnitine palmitoyltransferase 1 | 1.6000e+03 mM/h |
| $v_{gpat}$ | glycerol-3-phosphate acyltransferases | 5.4000e+02 mM/h |
| $v_{agpat}$ | acylglycerolphosphate acyltransferase | 5.4000e+02 mM/h |
| $v_{pap}$ | phosphatidate phosphatase-1 | 3.6000e+03 mM/h |
| $v_{CPT}$ | choline phosphotransfer | 3.6000e+00 mM/h |
| $v_{PC_{exp}}$ | Phosphatidylcholine export | 3.6000e+03 h^-1^ |
| $v_{ApoB}^{syn}$ | ApoB synthesis | 3.6000e+03 mM/h |
| $v_{ApoB}^{deg}$ | ApoB degradation | 2.8800e+04 h^-1^ |
| $v_{MTP}$ | Microsomal transfer protein | 1.080e+01 mM^-1^h^-1^ |
| $v_{VLDL_{ex}}$ | VLDL export | 5.7600e+01 mM/h |
| $v_{TAG_{ER}}^{DGAT1}$ | DGAT1 at ER | 2.8800e+02 mM/h |
| $v_{TAG_{ER2}}^{DGAT2}$ | DGAT2 at ER | 3.0240e+02 mM/h |
| $v_{{TAG}_{LD^{n}}}^{DGAT2}$ | DGAT2 at LD | 3.0240e+02 mM/h |
| $v_{TAG_{LD^{n}}}^{fill_{DGAT2}}$ | Change of lipid droplet TAG due to DGAT2 | - |
| $v_{{LD}_{1}}^{de novo}$ | Nascent lipid droplet synthesis1 | 3.6000e+00 h^-1^ |
| $v_{{LD}_{2}}^{de novo}$ | Nascent lipid droplet synthesis2 | 3.6000e+00 h^-1^ |
| $v_{on}^{Plin1}$ | Perilipin binding to LD | 1.8000e+04 h^-1^ |
| $v_{off}^{Plin1}$ | Perilipin dissociation from LD | 3.6000e+03 h^-1^ |
| $v_{on}^{ADRP}$ | ADRP binding to LD | 1.2600e+05 h^-1^ |
| $v_{off}^{ADRP}$ | ADRP dissociation from LD | 3.6000e+03 h^-1^ |
| $v_{on}^{TIP47}$ | TIP47 binding to LD | 3.6000e+03 h^-1^ |
| $v_{off}^{TIP47}$ | TIP47 dissociation from LD | 3.6000e+03 h^-1^ |
| $v_{LD_{1}}^{TIP47}$ | Nascent Lipid Droplet coating with Tip47 | 3.6000e+03 h^-1^ |
| $v_{on}^{CGI58}$ | CGI58 binding to LD | 3.6000e+03 h^-1^ |
| $v_{off}^{CGI58}$ | CGI58 dissociation from LD | 3.6000e+03 h^-1^ |
| $v_{{ATGL}_{LD}}^{on}$ | ATGL binding to LD | 3.6000 µm^-2^h^-1^ |
| $v_{{ATGL}_{LD}}^{off}$ | ATGL dissociation from LD | 3.6000e+01 h^-1^ |
| $v_{{ATGL}_{LD}}^{CGI58 on}$ | ATGL binding to CGI58 | 3.6000 µm^-2^h^-1^ |
| $v_{{ATGL}_{LD}}^{CGI58 off}$ | ATGL dissociation from CGI58 | 3.6000e+01 h^-1^ |
| $v_{ATGL_{LD}}$ | adipose triglyceride lipase | 7.920e+00 h^-1^ |
| $v_{ATGL}^{TAG^{nld}}$ |  | 3.600e+03 h^-1^ |
| $v_{HSL}^{TAG}$ | hormone sensitive lipase acting on LD TAG | 5.4000e-03  mM µm^-2^h^-1^ |
| $v_{HSL}^{DAG}$ | hormone sensitive lipase acting on LD DAG | 5.4000e+00 mM µm^-2^h^-1^ |
| $v_{MGL}$ | monoacylglycerol lipase | 3.6000e+03 mM µm^-2^h^-1^ |
| $v_{Tag_{LD^{n}}}^{fus}$ | Lipid droplet fusion | 1.8000e-02  mM µm-2h-1 |

References

1. Stremmel, W., G. Strohmeyer, and P.D. Berk, *Hepatocellular Uptake of Oleate Is Energy-Dependent, Sodium Linked, and Inhibited by an Antibody to a Hepatocyte Plasma-Membrane Fatty-Acid Binding-Protein.* Proceedings of the National Academy of Sciences of the United States of America, 1986. **83**(11): p. 3584-3588.

2. Kampf, J.P. and A.M. Kleinfeld, *Fatty acid transport in adipocytes monitored by imaging intracellular free fatty acid levels.* Journal of Biological Chemistry, 2004. **279**(34): p. 35775-35780.

3. Kim, J.H., T.M. Lewin, and R.A. Coleman, *Expression and characterization of recombinant rat Acyl-CoA synthetases 1, 4, and 5. Selective inhibition by triacsin C and thiazolidinediones.* J Biol Chem, 2001. **276**(27): p. 24667-73.

4. Saggerson, E.D., C.A. Carpenter, and B.S. Tselentis, *Effects of thyroidectomy and starvation on the activity and properties of hepatic carnitine palmitoyltransferase.* Biochem J, 1982. **208**(3): p. 667-72.

5. Saggerson, E.D. and C.A. Carpenter, *Effects of fasting and malonyl CoA on the kinetics of carnitine palmitoyltransferase and carnitine octanoyltransferase in intact rat liver mitochondria.* FEBS Lett, 1981. **132**(2): p. 166-8.

6. McGarry, J.D. and N.F. Brown, *The mitochondrial carnitine palmitoyltransferase system. From concept to molecular analysis.* Eur J Biochem, 1997. **244**(1): p. 1-14.

7. McGarry, J.D., et al., *Observations on the affinity for carnitine, and malonyl-CoA sensitivity, of carnitine palmitoyltransferase I in animal and human tissues. Demonstration of the presence of malonyl-CoA in non-hepatic tissues of the rat.* Biochem J, 1983. **214**(1): p. 21-8.

8. Yamashita, S. and S. Numa, *Partial purification and properties of glycerophosphate acyltransferase from rat liver. Formation of 1-acylglycerol 3-phosphate from sn-glycerol 3-phosphate and palmityl coenzyme A.* Eur J Biochem, 1972. **31**(3): p. 565-73.

9. Yada, R., H. Ide, and Y. Nakazawa, *In vitro effects of chlorpromazine on glycerol-3-phosphate acyl transferase and 1-acylglycerol-3-phosphate acyltransferase in rat liver microsomes.* Biochem Pharmacol, 1986. **35**(22): p. 4083-7.

10. Yamashita, A., et al., *Topology of acyltransferase motifs and substrate specificity and accessibility in 1-acyl-sn-glycero-3-phosphate acyltransferase 1.* Biochim Biophys Acta, 2007. **1771**(9): p. 1202-15.

11. Han, G.S. and G.M. Carman, *Characterization of the human LPIN1-encoded phosphatidate phosphatase isoforms.* J Biol Chem, 2010. **285**(19): p. 14628-38.

12. Morimoto, K. and H. Kanoh, *Acyl Chain-Length Dependency of Diacylglycerol Cholinephosphotransferase and Diacylglycerol Ethanolaminephosphotransferase - Effect of Different Saturated Fatty-Acids at C-1 or C-2 Position of Diacylglycerol on Solubilized Rat-Liver Microsomal-Enzymes.* Journal of Biological Chemistry, 1978. **253**(14): p. 5056-5060.

13. Durrington, P.N., et al., *Effects of insulin and glucose on very low density lipoprotein triglyceride secretion by cultured rat hepatocytes.* J Clin Invest, 1982. **70**(1): p. 63-73.

14. Coleman, R. and R.M. Bell, *Triacylglycerol synthesis in isolated fat cells. Studies on the microsomal diacylglycerol acyltransferase activity using ethanol-dispersed diacylglycerols.* J Biol Chem, 1976. **251**(15): p. 4537-43.

15. Hosaka, K., U. Schiele, and S. Numa, *Diacylglycerol acyltransferase from rat liver microsomes. Separation and acyl-donor specificity.* Eur J Biochem, 1977. **76**(1): p. 113-8.
